# Towards Clinical Translation of *In Situ* Cartilage Engineering Strategies: Optimizing the Critical Facets of a Cell-Laden Hydrogel Therapy

**DOI:** 10.1007/s13770-022-00487-9

**Published:** 2022-10-16

**Authors:** Serena Duchi, Sam L. Francis, Carmine Onofrillo, Cathal D. O’Connell, Peter Choong, Claudia Di Bella

**Affiliations:** 1 Department of Surgery, The University of Melbourne, Melbourne, VIC Australia; 2 BioFab3D-ACMD-St Vincent’s Hospital, Melbourne, VIC Australia; 3 Electrical and Biomedical Engineering, School of Engineering, RMIT University, Melbourne, VIC Australia; 4 Department of Orthopaedics, St Vincent’s Hospital, Melbourne, VIC Australia

**Keywords:** In situ cartilage engineering, Articular cartilage, Photocrosslinkable hydrogels, Infrapatellar fat pad, Mesenchymal stem cells

## Abstract

**Background:**

Articular cartilage repair using implantable photocrosslinkable hydrogels laden with chondrogenic cells, represents a promising *in situ* cartilage engineering approach for surgical treatment. The development of a surgical procedure requires a minimal viable product optimized for the clinical scenario. In our previous work we demonstrated how gelatin based photocrosslinkable hydrogels in combination with infrapatellar derived stem cells allow the production of neocartilage *in vitro*. In this study, we aim to optimize the critical facets of the *in situ* cartilage engineering therapy: the cell source, the cell isolation methodology, the cell expansion protocol, the cell number, and the delivery approach.

**Methods:**

We evaluated the impact of the critical facets of the cell-laden hydrogel therapy *in vitro* to define an optimized protocol that was then used in a rabbit model of cartilage repair. We performed cells counting and immunophenotype analyses, chondrogenic potential evaluation via immunostaining and gene expression, extrusion test analysis of the photocrosslinkable hydrogel, and clinical assessment of cartilage repair using macroscopic and microscopic scores.

**Results:**

We identified the adipose derived stem cells as the most chondrogenic cells source within the knee joint. We then devised a minimally manipulated stem cell isolation procedure that allows a chondrogenic population to be obtained in only 85 minutes. We found that cell expansion prior to chondrogenesis can be reduced to 5 days after the isolation procedure. We characterized that at least 5 million of cells/ml is needed in the photocrosslinkable hydrogel to successfully trigger the production of neocartilage. The maximum repairable defect was calculated based on the correlation between the number of cells retrievable with the rapid isolation followed by 5-day non-passaged expansion phase, and the minimum chondrogenic concentration in photocrosslinkable hydrogel. We next optimized the delivery parameters of the cell-laden hydrogel therapy. Finally, using the optimized procedure for *in situ* tissue engineering, we scored superior cartilage repair when compared to the gold standard microfracture approach.

**CONCLUSION:**

This study demonstrates the possibility to repair a critical size articular cartilage defect by means of a surgical streamlined procedure with optimized conditions.

**Supplementary Information:**

The online version contains supplementary material available at 10.1007/s13770-022-00487-9.

## Introduction

Tissue engineering strategies using chondrogenic cells and implantable photocrosslinkable hydrogels aim to offer a treatment strategy to repair articular cartilage defects, to ultimately delay the need for joint replacement surgery [1, 2].

The clinical translation of this approach requires technical optimisation, therapeutic efficiency verification and minimisation of costs. Importantly, key facets are essential for cartilage repair such as the optimal source of cells; the minimal timeframe to harvest and process those cells; the minimum concentration of cells required; which defect sizes are repairable in relation to cells number; the conditions to deliver preferred biomaterials into the defect.

The ideal cell type used for *in situ* cartilage engineering repair should be safely harvestable, minimally manipulated during isolation and expansion, and chondrogenic [3]. Sources of cells from within the human knee joint include adipose-derived stem cells (hADSCs), mature human chondrocytes (hCHOs) and articular progenitor cells (hAPCs) [4, 5]. Both chondrocytes and progenitors are isolated from articular cartilage of the non-weight bearing zones of the joint, where there is minimal tissue availability, low cell number and a risk of damaging the adjacent healthy weight-bearing tissue [6, 7]. hADSCs are isolated from the infrapatellar fat pad (IFP) of the knee, and can also be safely removed arthroscopically or with an arthrotomy [8–11]. An optimized isolation procedure where tissue breakdown and plastic adherence are limited, is required to obtain the maximum number of stem cells with the minimal *ex situ* manipulation [12, 13], avoiding exposure of the tissue to enzymatic treatment with associated risks of dysregulated cell function [14], cytotoxicity and pathogenicity [15, 16]. The literature suggests that collagenase digestion to retrieve cells from the tissue reduces their viability when these procedures take longer than 30 minutes [17]. Plastic adherence using non-coated tissue culture polystyrene plates takes at least 24 hours [18], while matrigel matrix [19] and fibronectin [20] as coating material can speed up adherence (within 30 minutes) [21] with no effect on the molecular characteristics [22].

Optimizing the cells expansion phase is crucial because lengthy laboratory-based expansion protocols lead to concerns with potential clinical application due to prolonged exposure to animal serum-based media, sterility, loss of differentiation potential, and tumorigenic transformation [23]. Therefore, the expansion phase can be maintained to a minimal standard only if a minimum number of cells required to repair a specific size defect is defined.

The delivery parameters into a cartilage defect also require optimization for the surgical application. Hydrogel materials provide a biocompatible and biodegradable 3D structure, analogous to cartilaginous extracellular matrix (ECM) [24]. In particular, photocrosslinkable hydrogels can be delivered into complex defect morphologies which can subsequently be solidified using a light based crosslinking process, and therefore, can be used for *in situ* delivery therapies [25]. The ideal photocrosslinkable hydrogel should be selected based on biocompatibility, gelation temperature, gelation time, while the light crosslinking parameters such as intensity, time and intraoperative device characteristics, must be compliant with the surgical scenario [26]. Among them, gelatine methacryloyl (GelMA) is naturally derived, it has been widely used in cartilage regeneration, and is approaching the clinical translation [27–29].

**Fig. 1:**
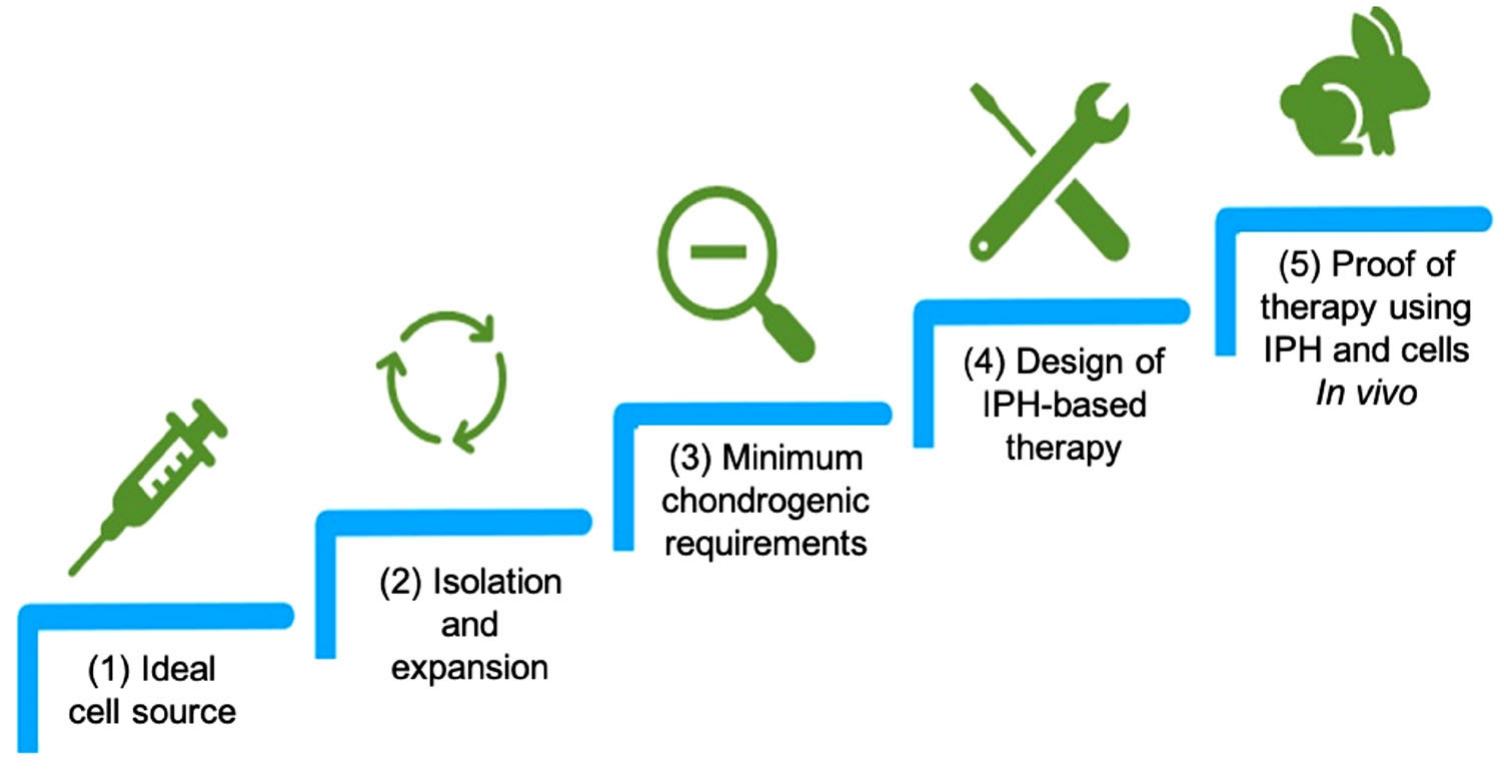
Research workflow and progression. In this study we (1) identified the most chondrogenic source of adult cells within the human knee; (2) developed a minimally manipulated cell retrieval and proliferation procedures; (3) determined the minimum cell expansion time frame and the minimum cell concentration in the photocrosslinkable hydrogel; (4) optimized the delivery parameters of the cell-laden injectable photocrosslinkable hydrogel (IPH); (5) evaluated the performance of the cell-laden injectable photocrosslinkable hydrogel (IPH) therapy *in vivo*

In this study we describe the key facets to optimize a cell-laden hydrogel therapy for the clinical translation (Fig. 1). We first identified the best chondrogenic cell source within the knee joint; we optimised the isolation procedure of hADSCs from IFP; we investigated the minimum timeframe required to obtain an optimal number of cells to produce neocartilage in a hydrogel *in vitro*; we tested the delivery parameters of the cell-laden hydrogel and the performance of the therapy *in vivo*.

## Materials and methods

### Cell isolation protocols and counting

Human IFP and articular cartilage were harvested from patients undergoing elective knee arthroplasty with informed consent (study approved by the Human Research Ethics Committee of St. Vincent's Hospital, Melbourne, Australia, HREC/16/SVHM/186). We isolated stem cells, chondrocytes, and progenitor cells from the same patients to compare the different cells type from the same donor source (n = 3 patients).

For the control hADSCs, which is based on our previous work [11], fresh IFP was placed on a sterile glass dish, and fat was mechanically dissociated from fibrous material, diced and then digested using 0.1% (1 mg ml^−1^ or 345U/ml) collagenase type II (Worthington Biochemical Corporation, Lakewood, NJ, USA) for 3 h at 37 °C under constant agitation (160 rpm). After a centrifugation step at 2100*g* for 10 min to separate the oil, fat and cells phase, the cells were filtered through a 100 µm cell strainer nylon (BD Falcon, Franklin Lakes, NJ, USA) and centrifuged at 400*g* at room temperature for 5 min to separate the stromal fraction from the floating adipocytes. The supernatant was discarded, and the cells were resuspended in Red Cell Lysis Buffer (160 mM NH4Cl; Sigma-Aldrich, St. Louis, MO, USA) and incubated at room temperature for 10 min. The cells were finally centrifuged at 400*g* at room temperature for 5 min and filtered through a 40 µm nylon cell strainer (BD Falcon). The initial cell count and viability were performed before cells were plated on non-coated tissue culture polystyrene (TCPS) and incubated for 24 h in complete hADSCs culture media [low glucose Dulbecco's modified eagle medium (DMEM) (Sigma-Aldrich) supplemented with 10% foetal bovine serum (Gibco, Thermo Fisher Scientific Inc., Waltham, MA, USA), 100 U ml^−1^ penicillin and 100 μl ml^−1^ streptomycin solution (Gibco), 2 mM l-glutamine (Gibco), and 15 mM HEPES (Gibco), 20 ng ml^−1^ epidermal growth factor and 1 ng ml^−1^ fibroblast growth factor (R&D Systems Inc., Minneapolis, MN, USA)]. The cell adherence percentage and hADSCs count was calculated before expansion.

For the rapid hADSCs isolation procedure, all steps were identical to the control isolation procedure detailed above, except from the following two changes: (1) chemical digestion was achieved in 30 min using 0.3 ml of 10 mg ml^−1^ collagenase type II (Worthington Biochemical, Lakewood, NJ, USA); (2) cells were plated on Matrigel-coated TCPS wells (Lifesciences, Corning, Tewksbury, MA, USA) and incubated for 30 min to allow for cellular adherence. Wells were coated as per following manufacturer’s protocol: 100X Matrigel (Sigma-Aldrich) was diluted in DMEM low glucose and 1 ml of solution was added to each desired well of a 6-well plate. The wells were air-dried for 1 h with the lid off under a biosafety hood, and the excess was then aspirated. The plates were used immediately or stored wrapped in parafilm at 4 °C for up to 7 days. The initial cell count and viability were performed before cells were plated on Matrigel, and the cell adherence percentage and hADSCs count was calculated before expansion.

The mature hCHOs and the articular progenitor cells (hAPCs) were isolated from cartilage with a macroscopically normal appearance that was excised from the femoral condyles of osteoarthritic patients undergoing total joint knee replacement surgery, as reported previously [30]. Cells were harvested from fresh cartilage pieces that were placed in a sterile glass petri dish and finely diced with a scalpel, rinsed with 1× PBS solution and then incubated in trypsin–EDTA 0.25% solution (Gibco) solution for 30 min at 37 °C under constant agitation (160 rpm). The trypsin was discarded, the tissue was washed with 1 × PBS and then digested with 1 mg ml^−1^/well collagenase 2 solution diluted in complete chondrocyte culture media [DMEM/HAMF 12 (Sigma-Aldrich) supplemented with 10% FBS, 100 U ml^−1^ penicillin and 100 μl ml^−1^ streptomycin solution, and 2 mM l-glutamine] and incubated at 37 °C on a rotating shaker at 160 rpm for 3 h and then 12 h at 100 rpm. The digested tissue was then centrifuged 1500*g* for 10 min, wash 2 times in 1 × PBS + Pen/Strep and centrifuged 1500*g* for 3 min. The pellet was resuspended in 5 ml of complete medium, filtered through a 100 µm cell strainer nylon (BD Falcon), centrifuged at 400*g* for 3 min. The supernatant was discarded, and the pellet resuspended in complete culture media: half of this solution was plated and expanded, while the second half was further diluted in complete hAPCs medium [low glucose DMEM supplemented with 10% FBS, 100 U ml^−1^ penicillin and 100 μl ml^−1^ streptomycin solution, 2 mM l-glutamine, and 15 mM HEPES]. hAPCs were then plated into fibronectin-coated (Sigma-Aldrich) TCPS-coated as per manufacturers protocol and incubated for 20 min at 37 °C [31]. Briefly**,** lyophilised fibronectin powder was reconstituted with 2 ml of sterile water (2 mg ml^−1^, 2000×), allowed to dissolve for 30 min at 37 °C and then diluted the to 10 mg ml^−1^ in sterile 1× PBS to coat the surface of cell culture plate with a minimal volume (i.e. 500 μl for well of a 6-well plate). Plates were air-dried for 45 min at room temperature under a biosafety hood with the lid off. The excess was aspirated, and then plates stored at 4 °C closed with parafilm until downline use. Non-attached cells were removed, and fresh culture media was added, enabling hAPCs expansion.

The cell count and cell viability for the three groups of cells were calculated with Trypan Blue Stain (0.4%) method for use with the Countess™ Automated Cell Counter (Thermo Fisher Scientific) following the manufacturer’s instructions.

The proliferation rate of the cells between isolation and passage (Table 2) was evaluated by calculating their doubling time over 7 days. The cell population doubling time was calculated using the following equation *Doubling time* = (*t*2 − *t*1) × [ln (2)/In (*n*2/*n*1)].

Theorem: Where *t*2–*t*1 is the number of days in culture, *n*2 is the number of cells recovered after the duration of expansion and *n*1 is the total number of cells seeded.

The percentage of adherence was calculated using the following equation:

$${\text{Adherence}}\;\left( \% \right) \, = \frac{{\left[ {\left( {{\text{Isolation cell count}}/2} \right) - {\text{non-attached cell count}}} \right] \times 100}}{{\left( {{\text{Isolation cell count}}/2} \right)}}$$

### Immunophenotyping

The immunophenotypic characterisation was performed using a fluorescence-activated cell sorting (FACS) analysis of cell-surface markers. Cells (passage 2) were labelled with monoclonal antibodies against CD31, CD34, CD45, CD73, CD90, CD106, CD146—FITC conjugate, CD105, CD29, CD44 and CD49c—APC conjugate (eBioscience, San Diego, CA, USA). Control samples were labelled with isotype-matched control antibodies IgG1K-FITC and IgG1K-APC (eBioscience). In brief, cells were trypsinised, aliquoted, fixed in 0.5% paraformaldehyde for 30 min at 4 °C and washed. Next, samples were incubated with either conjugated specific antibodies or isotype-matched control, diluted in 1× PBS supplemented with 5% FBS (FACS buffer). Labelled cells were washed, suspended in FACS buffer and analysed using a FC500 flow cytometer (Beckman Coulter, Lane Cove West NSW 2066, Australia).

### *In vitro* pellet chondrogenesis

Chondrogenic differentiation of hADSCs, hCHOs and hAPCs was induced using the micro mass pellet culture technique described previously [32, 33]. Briefly 2.5 × 10^5^ confluent cells (passage 3) ml^−1^ were placed in 1.5 ml tube, centrifuged for 5 min to form pellets and initially cultivated with complete culture media. Chondrogenic differentiation was commenced once the spheres were formed (3 days from centrifugation) using DMEM high-glucose, 100 U ml^−1^ penicillin and 100 μl ml^−1^ of streptomycin, 2 mM l-glutamine, 15 mM HEPES, 1% insulin-transferring-selenium (Sigma-Aldrich), 100 nM dexamethasone (Sigma-Aldrich), 50 mg ml^−1^ ascorbate-2-phosphate (Sigma-Aldrich), 10 ng ml^−1^ TGFβ3 (Prepotech, Rocky Hill, NJ, USA), and 10 ng ml^−1^ BMP6 (R&D Systems). Media was changed twice a week for a total of 3 weeks differentiation. The area of the pellets was calculated using ImageJ on brightfield images taken with a stereo microscope equipped with a CMSO APTINA COLOR camera 5.1 MP ½0.5’’.

For chondrogenic analysis, pelleted cells were fixed in 1% paraformaldehyde (Santa Cruz Biotechnology, Dallas, TX, USA) for 4 h at room temperature, embedded in O.C.T. TM Compound (Tissue-Tek, Sakura, Leiden, Netherlands) and flash frozen in liquid nitrogen. Cryosections of 10 µm thickness was mounted onto glass slides and stained with SafraninO (Sigma-Aldrich) for 10 min, dipped in 95% and 100% EtOH, cleared three times for 1 min each in Xylene (Chem-Supply, GILLMAN, SA, Australia) and then mounted in Pertex medium (Grale HDS, Ringwood, VIC, Australia).

### Generation of hydrogel bioscaffolds and chondrogenesis

To test the chondrogenic capacity of hADSCs-laden hydrogel in bioscaffolds, GelMA/hyaluronic acid (HA) was used and synthesised as previously described [34, 35]. The material was dissolved to a final concentration of GelMA 10%/HA 2.5% in sterile 1 × PBS and 0.05% lithium phenyl-2,4,6-trimethylbenzoylphosphinate (LAP, Tokyo Chemical Industries, Tokyo, Japan) was added as photoinitiator. Cells at passage 3 were directly mixed into the hydrogel to form 3 different concentrations: 1.25, 2.5 and 5.0 million hADSCs/ml. Disposable low dead volume 1 ml syringe (Henke Sass Wolfe, Tuttlingen, Germany) with an 840 µm inner diameter nozzle attached was used as a delivery device and the cell-laden hydrogel was casted into 200 μl volume PDMS cylindrical moulds of 10 mm diameter and 2 mm thickness. The samples were irradiated at room temperature for 10 s, using a 365 nm light source (Omnicure LX400+, Lumen DynamixLDGI) with an intensity of 700 mW/cm^2^. Bioscaffolds were cultivated in chondrogenic differentiation media as described above and media was changed twice a week for a total of 3 weeks differentiation.

### RNA extraction reverse transcription and qPCR

RNA was extracted and purified from cells pellets and bioscaffolds using the Direct Trizol-RNA miniprep kit (Zymo Research) as per the manufacturer's protocol. The RNA concentration and purity were measured using Clariostar Plate Reader (BMG Biotech). 200 ng of total RNA was reverse transcribed into cDNA using High-Capacity Reverse transcription kit (Thermo Scientific) as per the manufacturer's protocol. TaqMan Gene expression assay (Applied Biosystems) was used to evaluate the relative expression of chondrogenic markers using the following probes: SOX9 (Hs00165814_m1), aggrecan (ACAN) (Hs00153936_m1), COL2A1 (Hs00264051_m1), collagen type 1 (COL1) A2 (Hs01028956_m1) and GAPDH (Hs02786624_g1) as the housekeeping gene. qPCR was performed using Quant Studio 6 Flex Real-Time PCR System (Thermo Fisher Scientific). Relative quantification was calculated with the 2e^−ΔΔCT^ method. The mean ΔCT value of the control sample was used in each experiment to calculate the ΔΔCT value of sample replicates by using the housekeeping gene (GAPDH).

### Glycosaminoglycans (GAG) and DNA content quantification

For GAG/DNA quantification, cell pellets and bioscaffolds were collected, digested for 5 h at 60 °C using papain extraction solution: 0.2 M Sodium Phosphate buffer, 0.01 M Cysteine, 0.2 M NaH_2_PO_4_ monohydrate, 0.01 M EDTA C_10_H_14_N_2_Na_2_O_8_·2H_2_O, 250 μg ml^−1^ papain, (ROCHE #10108014001, 30 U/mg). The lysate was then centrifuged at 10.000*g* for 10 min and supernatant aliquots were separately assayed for GAG and DNA content. GAG content was determined using the dimethylmethylene blue (DMMB) method using chondroitin sulphate as standard. 3.2 mg of DMMB were diluted in 0.6 g glycine, 0.32 g NaCl and 19 ml of 0.1 M acetic acid. DNA content was determined with Quant-iT PicoGreen dsDNA Assay kit (Invitrogen, Thermo Fisher Scientific Inc., Waltham, MA, USA). The GAG activity was calculated using a normalised GAG/DNA ratio.

### Staining analyses and imaging

Cells pellets and bioscaffolds were washed, fixed in 1% paraformaldehyde (Santa Cruz Biotechnology) for 4 h and then washed 3 times for 10 min in 1 × PBS. Next, samples were embedded in Sucrose 30%-dH_2_O overnight at 4 °C, embedded in OCT TM Compound (Tissue-Tek) and flash-frozen in liquid nitrogen. For fluorescence analysis, 10 μl thickness cryosections were washed and permeabilised for 15 min in 1 × PBS-0.1% TritonX-100 (PBT). Antigen retrieval was performed using Hyaluronidase (Sigma-Aldrich, #H6254) and incubated for 30 min at room temperature. After washing, samples were dropped in blocking solution (10% goat serum diluted in PBT) for 60 min and then incubated overnight at 4 °C with mouse anti-human Collagen type 2 (Col2) (#II6B3, DSHB) and goat anti-human Collagen type 1 (Col1) (# sc8784, Santa Cruz Biotechnology). The day after, samples were washed, and the secondary antibody anti-mouse IgG Alexa Fluor-647 (#715-605-151, Jackson Immuno Research, West Grove, PA, USA) and anti-goat IgG Alexa Fluor-546 (# A11056, Thermo Fisher Scientific Inc) were added and incubated for 2 h. After washing, actin was labelled with Phalloidin FITC (#P5282 Sigma-Aldrich) for 60 min; next nuclei were stained with DAPI (Thermo Fisher Scientific) for 60 min. Sections were washed, mounted onto glass slides and imaged with a NikonA1R confocal microscope and processed using NIS-Elements software (Nikon, Tokyo, Japan).

For SafraninO/Hematoxilin staining of pellet samples, cryosections were dipped in 100%, 95% and 80% EtOH, rinsed in dH_2_O, incubated in Weigert’s Haematoxylin (Sigma-Aldrich) for 5 min, rinsed in tap water, differentiated in Acid alcohol 1% (v/v) for 2 s, rinsed in dH_2_0 3 times, stained with SafraninO (Sigma-Aldrich) for 30 min, dipped in 95% and 100% EtOH, cleared three times for 1 min each in Xylene (Chem-Supply, GILLMAN, SA, Australia) and then mounted in Pertex medium (Grale HDS, Ringwood, VIC, Australia). Samples were imaged using an epifluorescent inverted NikonTiE microscope equipped with a DSRi2 and NIS-Elements software using a Plan Fluor ELWD 10X DIC L NA 0.45 objective. Figure panels were assembled using Photoshop software (Adobe). DAPI is represented in white and Phalloidin FITC in red in the Figs. 4 and 5.

### Rabbit *in vivo* study

This study was approved by the Animal Ethics Committee of St. Vincent's Hospital, Melbourne, Australia [AEC/002/19-r1]. Six New Zealand white male rabbits (Flinders, South Australia) 3 months of age (weight 2.7–3.0 kg) were sourced, acclimatised and individually housed in cages. General anaesthesia was induced using 35 mg/kg of ketamine and 5 mg/kg of xylazine and maintained intraoperatively using isoflurane/oxygen. A midline longitudinal incision was made over the knee followed by a medial parapatellar arthrotomy to access the joint. The patella was dislocated, and the IFP was removed before knee flexion to expose the femoral condyles. A central cylindrical full chondral defect was created using a 4 mm diameter biopsy punch leaving the subchondral bone untouched. The same procedure was performed in the contralateral knee. Animals were randomly allocated to groups and treatment groups was evenly assigned to medial and lateral condyles. The three treatment groups performed were: Empty defect → No repair performed; Microfracture → Subchondral bone pierced with a micro awl 3 times with bleeding observed; Therapy → 5.0 million/ml rabbit ADSCs (OriCell, Cyagen-GUXMX-90011) laden in hydrogel (described in paragraph 2.3) and loaded into a 1 ml pneumatic syringe, then placed in a 4 °C fridge for 3 min before being injected into the defect. After the delivery, the cell-laden hydrogel construct was hardened upon photocrosslinking (parameters described in paragraph 2.7) for 60 s. The patella was then reduced, and the joint capsules and skin were closed using 4–0 dexan and 5–0 monocryl sutures (Ethicon) respectively. Postoperative analgesia was subcutaneously administered as required and animals recovered on a heat pad before being returned to their pens and allowed to freely mobilise with daily monitoring. After 8 weeks, rabbits were humanely euthanised using inhalation anaesthesia followed by intravenous injection of lethobarb (Virbac Australia). The knee joint was exposed, disarticulated, and the femoral condyles were harvested. The defects were macroscopically assessed by four blinded investigators using the International Cartilage Repair Society (ICRS) score [36] (*Macroscopic evaluation*). Osteochondral blocks (1 by 1 cm) were then cut out using a handheld rotary saw (Dremel), with the treated defects positioned in the centre, allowing for 3 mm margins around the defect. Atomic force microscopy (AFM) nanoindentation was performed to evaluate the mechanical properties of the cartilage samples while immersed in PBS solution [37]. An MFP-3D origin (Asylum Research, Santa Barbara, CA, USA) AFM was used with a contact mode MLCT probe (Bruker Nano Inc). Force curves were presented to an indentation force of 5 nN at an approach rate of 2 μm s^−1^. The sample Poisson's ratio was set at 0.31 based on literature describing the ratio in 4-month-old male New Zealand white rabbits [38]. The young's modulus was obtained using the Hertz indentation model approximating the tip-shape as a 19.2° cone. Indentations (n = 100) across three regions were performed in a standardised fashion (*Mechanical evaluation*). The blocks were then fixed in 10% neutral buffered formalin (Sigma-Aldrich) for, decalcified using ETDA as previously described [39] [40], then embedded in paraffin wax. Cryosections of 7 μm thickness were mounted onto glass slides, stained with haematoxylin and eosin (H&E) and imaged using a high-resolution Mirax digital slide scanner. Images were processed using case viewer 2.3 software (3D Histotech, Budapest, Hungary). Fluorescence analysis was performed as described in paragraph 2.7, using anti-mouse Col2 (paragraph 2.7) and goat anti-rabbit Col1 (# UNLB, Southern Biotech) with the secondary antibody anti-goat IgG Alexa Fluor-546. The fluorescence measurement analysis of Fig. 6 was performed using ImageJ software’ colour threshold plugin. The positive area of the indicated staining was calculated as a percentage of the total area of 4 different region of interest (ROI) identified in the images. The different ROI were selected over the entire field of view, so the entire cross-section area of the immunostained sections was evaluated. Those analyses were performed on three different samples per condition.

Stained H&E, anti-Col1 and anti-Col2 samples were scored by three blinded investigators from different backgrounds (Molecular biologist, Cellular biologist and Surgeon) using a semiquantitative score [41] (*Microscopic evaluation*).

### Statistical analysis

All statistical analyses were performed using Prism 8 (GraphPad) software. Differences between the experimental groups were determined using the unpaired t-test. Significance was represented as follows:

$$* = p \, \le \, 0.05;\;** = p \, \le \, 0.01;\;*** = \, p \, \le \, 0.001;\;{\text{not significant}}\;\left( {\text{n.s.}} \right) = p > 0.05.$$

Unless otherwise stated, data are presented as mean ± standard error margin (SEM).

## Results

In the following results section, we present the experimental procedures performed to characterize the following critical facets of the cell-laden hydrogel therapy for cartilage repair: Cell Source, Isolation of chondrogenic cells, Expansion of chondrogenic cells, Cell concentration and Delivery.

### Cell source: comparative analysis identifies the infrapatellar fat pad as the source of cells with chondrogenic potential

**Fig. 2:**
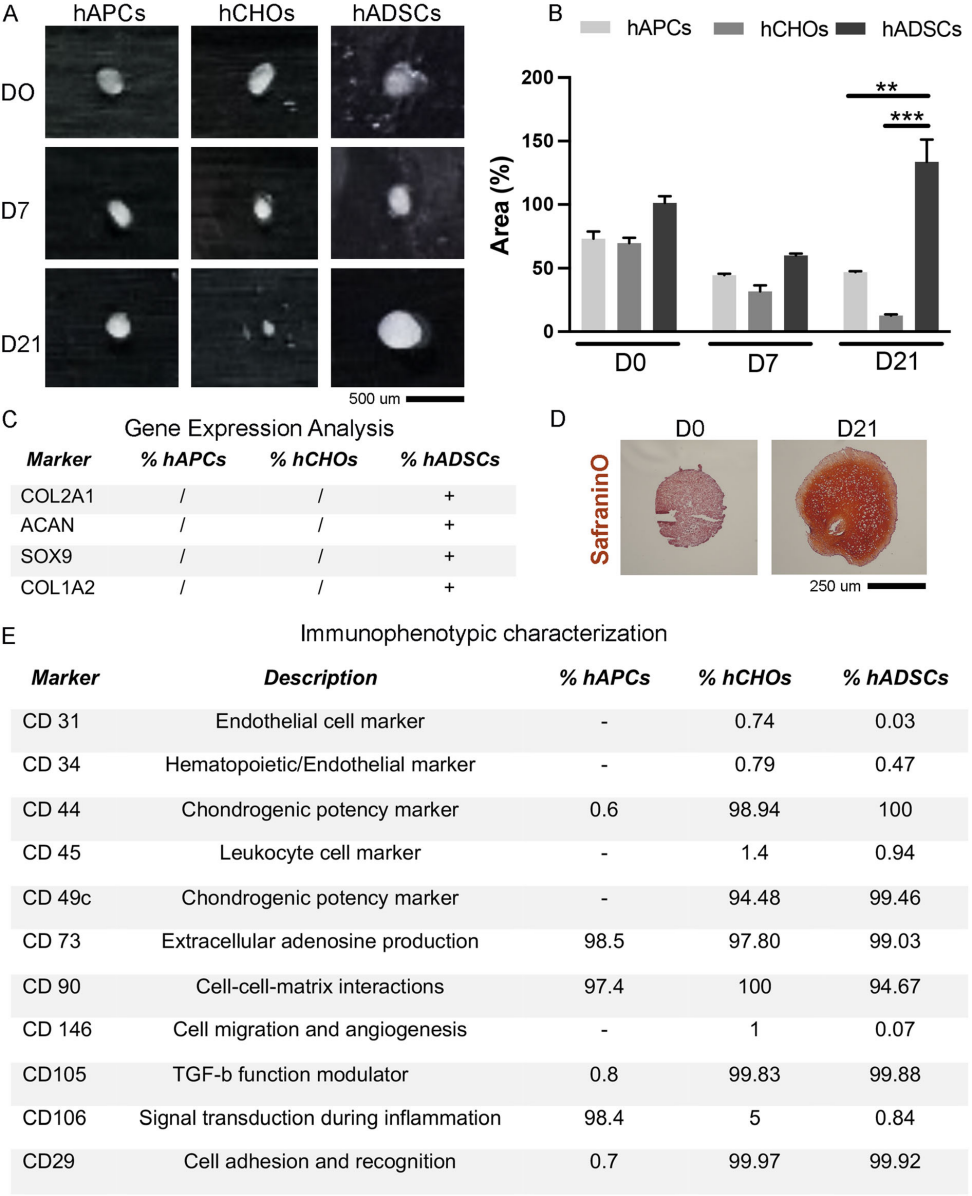
Chondrogenic comparison of hAPCs, hCHOs and hADSCs cell lines. Pellet cultures were generated from the three different cell lines isolated obtained from the same patient (n = 3 patients), and analyses performed at 3 different time points of chondrogenic differentiation (day 0-DO, day 7-D7, day21-D21). **A** Morphological comparison of pellet culture: representative macroscopic images of pellets generated with the 3 different cell types. **B** The graph shows the quantification of the individual areas of the samples generated with the 3 cell types expressed as percentage (%) normalized to hADSCs at day 0. **C** Chondrogenic gene expression analysis calculated among three biological replicates. The / and + indicate the absence or presence of detectable ct levels of the gene of interest: Collagen type 2A1 (COL2A1), Aggrecan (ACAN), Sox9 (SOX9) and Collagen type 1A2 (COL1A2) markers in RT-qPCR assay. GAPDH was used as the housekeeping gene and data were normalized against hADSCs at day 21. **D** Histological analysis on 10 µm cryosections stained with SafraninO to detect accumulation of GAG in the ECM. Representative brightfield images from hADSCs pellet at day 0 (D0) and day 21 (D21) of chondrogenic differentiation. **E** Immunophenotypic characterization: the table shows the cytofluorimetric analyses of the stemness markers used to characterize the three different cell lines harvested from the tissues belonging to the same donor knee. The most representative patient’s analysis is reported here (n = 3). *hAPCs* human articular progenitor cells, *hCHOs* human mature chondrocytes, *hADSCs* human adipose-derived stem cells

hAPCs, hCHOs and hADSCs, were harvested from tissues belonging to the same donor’s knee which were resected during arthroplasty. The three cell lines were isolated and culture-expanded for 3 passages before being induced to a 21 days chondrogenic differentiation in pellet culture (Fig. 2A–C). After 7 days (D7), the three cell populations showed a reduction in the area size attributable to cell condensation during the initial phases of the chondrogenesis process [42]. At day 21 (D21), the area of the hCHOs pellets continued to decrease and the size of the hAPCs remained constant, while the hADSCs pellets showed a significant increase in size (Fig. 2A, B). The gene expression of the chondrogenic markers performed on RNA extracted from the pellet cultures was consistent with the morphological analysis of the areas (Fig. 2C). In fact, while the housekeeping gene coding for Glyceraldehyde 3-phosphate dehydrogenase (GAPDH) was expressed in all the three groups of cells, the gene expression of COL2A1, ACAN, SOX9 and COL1A2 was undetectable in hCHOs and hAPCs when normalized to hADSCs at 21 days (Fig. 2C). The stem cells showed a detectable expression of Collagen type 2 and SOX9 only after the chondrogenic stimulation, associated with increase in ACAN and COL1A2 expression levels. Consistently, the extracellular matrix was visible only in the hADSC pellet at day 21 (Fig. 2D), while no extracellular matrix was detectable in the hCHOs and hAPCs pellets. SafraninO staining showed accumulation of Glycosaminoglycans (GAG) deposition and changes in cell morphology from round shapes at day 0 (D0) to elongated cells encapsulated by an abundant ECM in the entire pellet structure.

The data obtained from the immunophenotypic analysis performed on the three populations showed that CD44 (chondrogenic potency marker) was found to be present in a small fraction of the hAPCs population (0.6%), while 98.94% of the hCHOs and 100% of the hADSCs were positive. CD105 (TGF-β transduction) was expressed in the 0.8% of the hAPCs, while 99.83% of the hCHOs and 99.88% of the hADSCs populations were characterized by the presence of the marker. The signal transduction pathway during inflammation detected via CD106 where the least expressed in the hADSCs population (0.84%) compared to hCHOs (5%) and hAPCs (98.4%) (Fig. 2E).

Overall, hADSCs displayed the most chondrogenic potential, therefore were considered the optimal cell type to be used in the next phases of the study.

### Cell isolation: hADSCs can be efficiently isolated using a rapid procedure

**Fig. 3:**
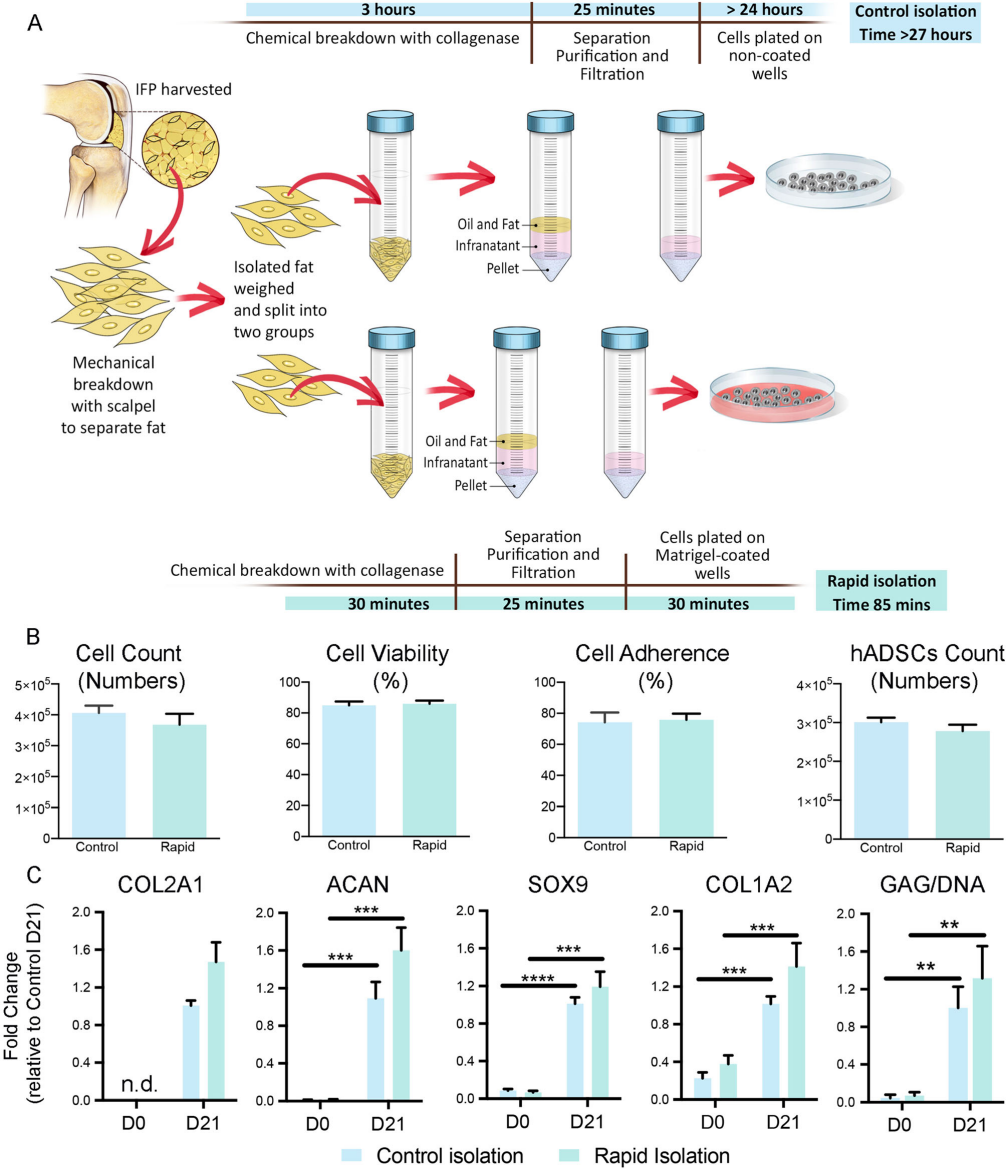
Rapid isolation procedure of hADSCs. **A** Graphical representation of the two protocols tested for hADSCs isolation. **B** The bar graphs represent the evaluation of cell retrieval ability divided per number of cells isolated (Cell Count), percentage of viability, percentage of adherence to the plastic and matrigel substrate, and number of hADSCs isolated (hADSCs Count). **C** Chondrogenic gene expression and Glycosaminoglycan assays: the bar graphs represent the fold changes calculated with 2^ΔΔCΤ^ method of Collagen type 2A1 (COL2A1), Aggrecan (ACAN), Sox9 (SOX9) and Collagen type 1A2 (COL1A2) markers in RT-qPCR assay. n.d. = not detectable. GAPDH was used as the housekeeping gene, and data were normalized to hADSCs at day 21. Glycosaminoglycan (GAG) content measured via the normalisation of GAG over total DNA present in the processed scaffolds. Graph bars represent standard error margin between three biological replicates. The two time points of the analysis (day 0-DO and day 21-D21) are reported in × axes of the graphs. Statistical analysis was performed using an unpaired t-test

We optimized the hADSCs isolation procedure from IPFs to avoid any unnecessary exposure of the tissue to enzymatic treatment, while increasing the recovery rate of the stem cell population and maintaining the chondrogenic differentiation potential. Compared to the isolation timeframe of the IFP based on our previous work, here referred as Control [11], the enzymatic breakdown step was reduced from 3 h to 30 min, and the plastic adherence phase was cut down to 30 min by using Matrigel-coated tissue culture plates to induce a faster and more efficient cells attachment (Fig. 3A). Of note, the control isolation is based on our protocol which was set up to isolate a chondrogenic population of the stem cells capable to undergo chondrogenic differentiation in pellet culture and in hydrogel bioscaffolds.

The effect of the enzymatic digestion on the overall cell count and cell viability was evaluated at different time points (10, 20 and 30 min), and the 30 min was sufficient to obtain several cells comparable to the standard 3 h’ time frame (Supplementary Fig. S1). Next, to quantify the selective Matrigel-coating for adherence of hADSCs, the attachment and post-attachment cell count was evaluated at different time points compared to the non-coated plastic culture plates as the control group (Supplementary Fig. S2). At 30 min the percentage of cell adhesion reached the maximum level, and it was comparable to the standard 24 h’ time frame on non-coated plastic surface.

To evaluate this newly devised isolation protocol (defined as Rapid isolation), IFPs from three different patients were isolated, each fat pad was divided equally into two, with cell isolation performed using either rapid or standard (Control isolation) procedures (complete workflow shown in Fig. 3A). Retrieval efficiency, stemness, chondrogenic gene expression analysis and glycosaminoglycan accumulation were then evaluated for both protocols.

The rapid isolation approach yielded to 3.68 × 10^5^ (± 3.55 × 10^4^) compared to 4.07 × 10^5^ (± 2.32 × 10^4^) live cells in the control isolation approach with no significant difference measured (Fig. 3B, Cell Count). Mean cell viability was 86.04% (± 1.3) and 85.02% (± 1.42) for the rapid and control isolation groups, respectively, with no significant difference (Fig. 3B, Cell Viability). Mean cell attachment was 75.79% (± 2.31) in the rapid isolation group and 74.28% (± 3.63) in the control isolation group, with no significant difference (Fig. 3B, Cell Adherence). The number of hADSCs isolated post selective adherence in the rapid and control isolation groups was 2.79 × 10^5^ (± 6.81 × 10^3^) and 3.02 × 10^5^ (± 9.28 × 10^3^) respectively, with no significant difference (Fig. 3B, hADSCs Count). In all measures, no variations were detected between the two isolation groups; therefore, no alteration in the ability to isolate the hADSCs population by reducing the enzymatic breakdown duration and using Matrigel-coating for adherence was observed. Cells from both groups were then evaluated for their immunophenotypic fingerprint, and a flow cytometric analyses of cell surface markers was performed. Both groups expressed the expected hADSCs profile [> 90% positivity in CD44/49c/73/90, and < 3% positivity in CD31/34/45/146], with no significant difference (Table 1, Supplementary Fig. S3), proving no effect on phenotype by reducing the enzymatic digestion and using Matrigel-coating for adherence.

**Table 1:** Immunophenotypic analysis

Marker	hADSCs Phenotype	Description	Control %	Rapid %	*P* value
CD31	−	Endothelial cell marker	0.66	0.83	0.60
CD34	−	Hematopoietic/endothelial marker	2.24	2.79	0.68
CD44	+	Chondrogenic potency marker	99.86	99.82	0.57
CD45	−	Leukocyte cell marker	1.05	0.89	0.53
CD49c	+	Chondrogenic potency marker	95.43	95.52	0.98
CD73	+	Extracellular adenosine production	98.61	98.34	0.79
CD90	+	Cell–cell–matrix interactions	97.78	97.86	0.95
CD146	−	Cell migration and angiogenesis	2.28	1.92	0.80

The table shows the summary of the immunophenotype performed using flow cytometry on hADSCs isolated under control and rapid procedures. The most representative patient’s analysis is reported here (n = 3)

We then performed a 21 days chondrogenic differentiation study in pellet culture using cells obtained from both isolation groups. Gene transcription analyses revealed detectable levels of COL2A1 only after 21 days in both groups. ACAN, SOX9 and COL1A2 significantly increase after 3 weeks from the start of the chondrogenic stimulation (Fig. 3C). No significant difference was evident comparing chondrogenic differentiation between the rapid and control groups, suggesting no alteration in cell potency secondary to the modifications made to obtain the rapid isolation workflow. The increase in Collagen type 1 expression level was not correlated with a surge in the production and accumulation of the protein level as observed in our previous work [43] and as verified in Supplementary Fig. S4. The chondrogenic capacity of hADSCs isolated from both procedures, was also confirmed by the accumulation of GAG. In fact, the GAG content, normalized for the DNA amount, significantly increased in both rapid and control groups (Fig. 3C).

Both osteogenic and adipogenic differentiations were performed with cells isolated using the two isolation procedures, to confirm the trilineage capacity associated with a mesenchymal stem cell line. Osteogenic and adipogenic differentiation studies were conducted for 21 days using 2D culture of cells obtained in both isolation groups, revealing no significative differences in the differentiation capacity of both cell groups (Supplementary Fig. S5. 6).

### Cell expansion: hADSCs undergo chondrogenesis after minimal non-passaged culture

**Fig. 4:**
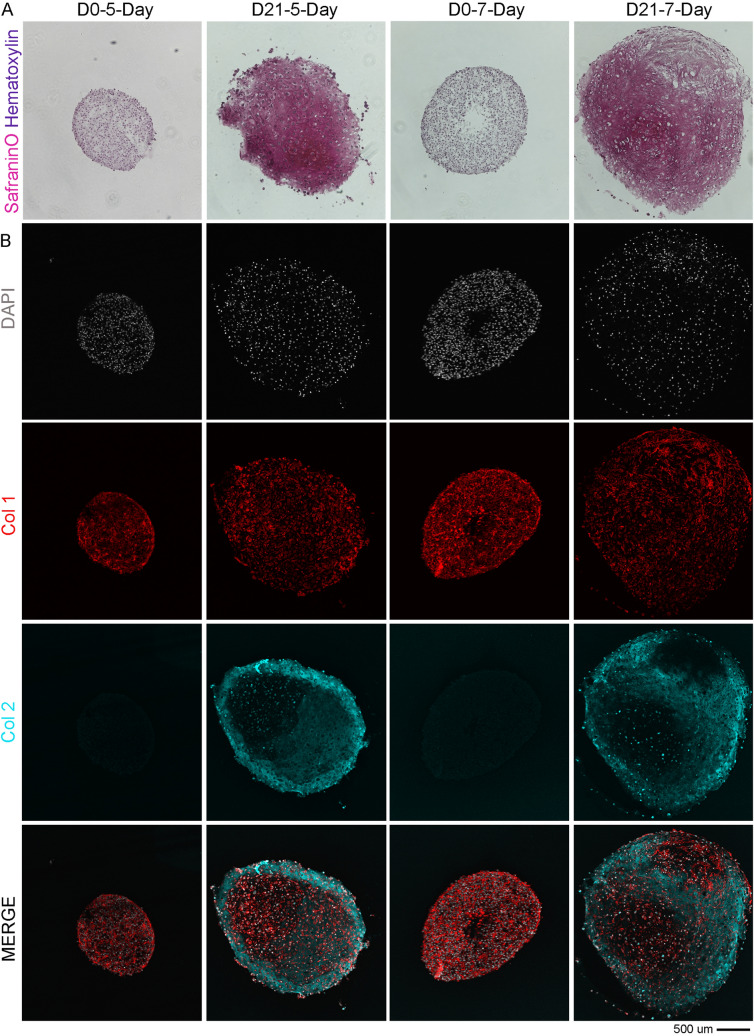
Minimum expansion time frame to obtain chondrogenic hADSCs from the isolation phase. **A** Representative brightfield images of cryosections from pellet culture stained with SafraninO (in pink) and Haematoxylin (in purple) to identify cell’s nuclei. **B** Representative confocal images of cryosections from pellet immunostained with Phalloidin-RFP (Actin, in red), Collagen type 2 (Col 2, in cyan) and counterstained to detect cells nuclei (DAPI, in white). Superimposed channels are shown in the last row of panels (MERGE). For all the stainings, the cryosections were obtained from cells pelleted after 5 and 7 days of non-passaged proliferation, pushed into 3 weeks of chondrogenic differentiation. The calculated Collagen type 2 intensity from day 0 to day 21 in reported as Fold Increase (F.I.) in the day 21 panels of their corresponding groups

To identify the minimal expansion phase after the isolation that allows the stem cells to successfully trigger chondrogenic differentiation, rapidly isolated hADSCs without further passaging from the last step of the isolation procedure, were immediately directed toward the chondrogenic differentiation in pellet cultures (Post Isolation > 0-Day) or expanded in proliferation media for additional 3, 5 and 7 days (Expanded > 3-Day, 5-Day, 7-Day) prior to the chondrogenic differentiation in pellet culture (Table 2). Only cells used after 5 and 7 days of no-passaged expansion, with an average of respectively 1.9 × 10^6^ and 2.8 × 10^6^ total cells, were able to successfully form spheroidal masses (Table 2). Pellets from both 5- and 7-Day time points grew significantly in size over 3 weeks of chondrogenic differentiation, suggesting an increase in ECM production as evidenced by SafraninO staining (Fig. 4A). As detected by immunostaining analyses, the Collagen type 2 (Col2), the main collagen component present in hyaline like cartilage, was significantly produced and released in both groups after 21 days of differentiation with no significant differences among the two groups (Fig. 4B). The cells, visualized by DAPI nuclear staining, from a compact distribution at day 0, progressively expanded their localization at day 21 as also observed by their morphology. The Phalloidin staining used to detect actin filaments, clearly show the shift from round shapes at day 0 (D0) to elongated and more complex phenotypes at day 21, as expected and already observed in chondrogenic differentiation of hADSCs. As reported in previous studies, human mesenchymal stem cells can be specifically primed for subsequent chondrogenic differentiation and ECM formation by stimulating cells with FGF2 during the expansion phase [44, 45]. Our hADSCs are cultivated and primed in a culture media containing EGF and FGF growth factors, which indeed promote the stem cells expansion and upregulates the transcription factor Sox9, critical in the early phases of chondrogenic differentiation of mesenchymal precursors [46]. Therefore, our data can be explained by the fact that only the cells used after 5 and 7 days of non-passaged expansion received the stimulatory effect of FGF necessary to induce proliferation and drive their chondrogenic potential once stimulated by chondrogenic growth factors. The calculated Collagen 2 intensity for the 2 conditions normalized for the area, resulted in a 6.1 fold increase from day 0 to day 21 for the 5-day, and 5.78 for the 7-Day. Therefore the 5-day non passage expansion phase was selected as the minimum time frame for hADSCs to undergo chondrogenesis after the rapid isolation step.

**Table 2:** Minimum expansion timeframe

Groups tested	Number of samples	Average number of hADSCs	Average doubling time (days)	Pellet formation
POST ISOLATION > 0-day	3	7.7 × 10^5^	–	−
Expanded > 3-day	3	1.57 × 10^6^	3.18	−
Expanded > 5-day	3	1.90 × 10^6^	6.56	+
Expanded > 7-day	3	2.80 × 10^6^	3.79	+

The table summarize the capacity to form pellet cultures after the indicated non-passaged expansion phases. The most representative patient’s analysis is reported here (n = 3)

### Cell concentration: evaluation of the hADSCs concentration to produce neocartilage formation in hydrogel bioscaffolds

**Fig. 5:**
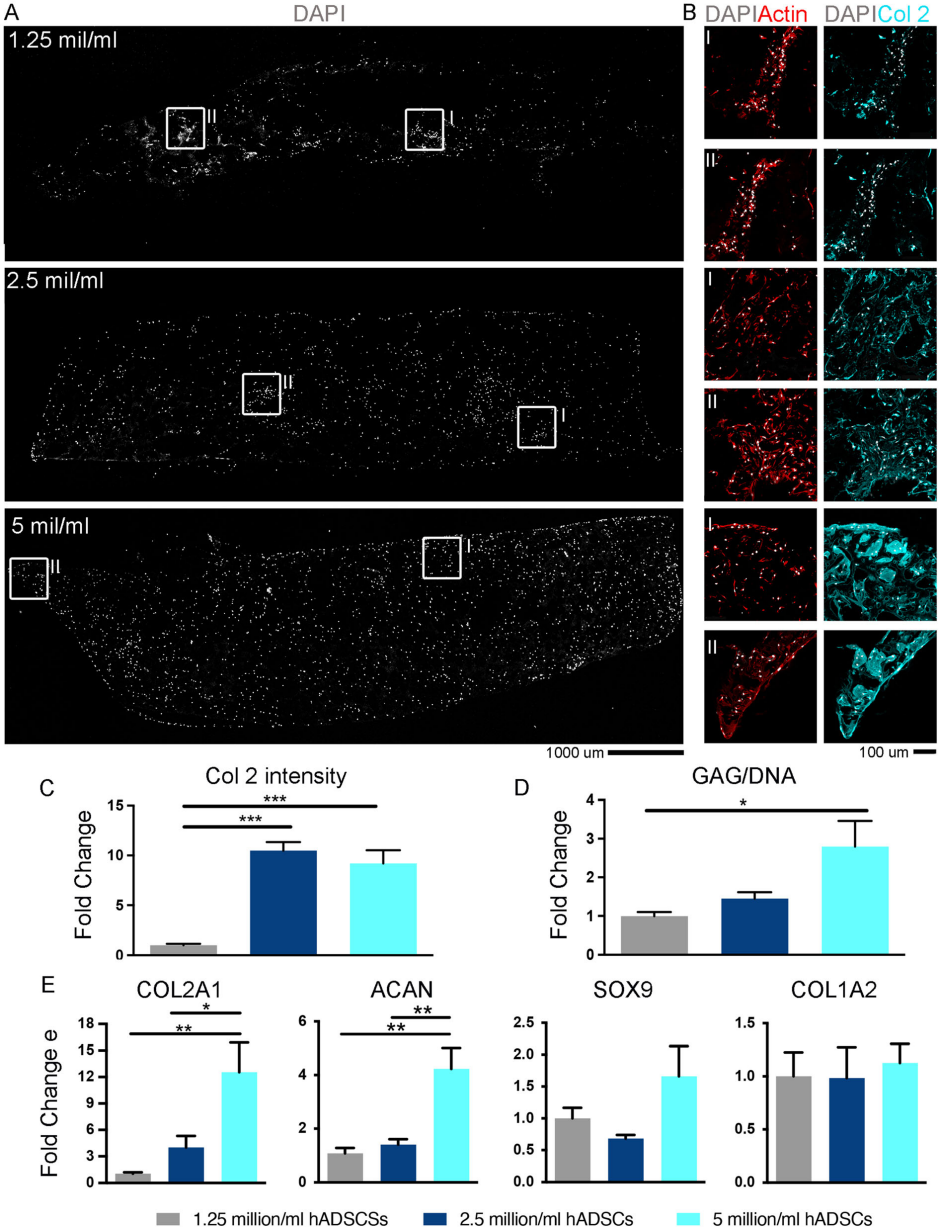
Assessment of minimal hADSCs concentration required for chondrogenesis in cell-laden hydrogel bioscaffolds. **A** Representative confocal images of cryosections from bioscaffolds from the 3 different hADSCs/ml groups, assessed using immunostaining for DAPI, Actin and Collagen type 2 (Col 2). The cryosections has been obtained by cutting the samples along the z axis to provide spatial information from the top to the bottom of the bioscaffolds. **B** Superimposed high magnification of the selected white square areas (I and II) in (**A**). **C** The graphs show the quantification expressed as fold change relative to 1.25 hADCSs/ml group at day 21 post chondrogenesis. The Collagen type 2 (Col 2) intensity signal was calculated and averaged from 16 different ROI from the Collagen II stained cryosections (see Figure S7). **D** The graphs show the quantification of Glycosaminoglycan (GAG) content measured via the normalisation of GAG over total DNA present in the processed bioscaffolds and expressed as fold change relative to 1.25 hADSCs/ml group at day 21 post chondrogenesis. **E** Chondrogenic gene expression analysis: the bar graphs represent the fold changes calculated with 2^ΔΔCΤ^ method of Collagen type 2A1 (COL2A1), Aggrecan (ACAN), Sox9 (SOX9) and Collagen type 1A2 (COL1A2) markers in RT-qPCR assay, relative to 1.25 hADSCs/ml group at day 21 post chondrogenesis. GAPDH was used as the housekeeping gene. Graph bars represent standard error margin between three biological replicates. Statistical analysis was performed using an unpaired t-test

To verify the minimum concentration of cells able to trigger neocartilage formation in hydrogels, rapidly isolated hADSCs were embedded in GelMA/HA at different concentrations (1.25, 2.5 and 5.0 million cells/ml), casted in cylindrical moulds and photocrosslinked to generate hydrogel bioscaffolds (for details see Materials and Methods section). A 21 days chondrogenic differentiation study was then performed to evaluate the minimum required concentration to produce hyaline extracellular matrix. Immunostainings, GAG accumulation and chondrogenic gene expression analyses were performed to evaluate the degree of neocartilage formation in the hydrogel (Fig. 5 and Supplementary Fig. S7). Cell density (DAPI, Fig. 5A) was found to be proportional with the concentration of hADSCs embedded in the hydrogel. The amount of Collagen type 2 (Col 2) production in the 2.5 million and the 5 million hADSCs/ml groups were respectively 10.5 and 9.2 times higher than the 1 million hADSCs/ml group (Fig. 5B). Despite the similar level of accumulation, Col 2 was mostly intracellular in the 2.5 million hADSCs/ml group, while in comparison more extracellular accumulation of the protein was visible in the 5.0 million hADSCs/ml group, indicating greater efficiency in the building of new extracellular matrix. After 21 days, the Collagen type 2 intensity was significantly higher at both 2.5 and 5 million groups (Fig. 5C and Supplementary Fig. S8), while the GAG content was significantly higher in the 5.0 million compared to 1.25 million hADSCs/ml groups (Fig. 5D). COL2A1 and ACAN gene expression were significantly higher in the 5.0 million hADSCs/ml group with a higher trend in SOX9 expression also noted (Fig. 5E), while COL1A2 remain unchanged among the 3 groups. This analysis overall demonstrates that adequate neocartilage formation can be achieved *in vitro* in cell-laden hydrogel bioscaffolds with a minimum of 5.0 million hADSCs/ml.

To identify the maximum repairable articular cartilage defect, the number of hADSCs reachable after the rapid isolation and the 5-Day non-passaged expansion phase from 1 or 2 fat pads, was correlated with the minimum chondrogenic hADSCs/ml concentration required to achieve neocartilage formation *in vitro*. Using these isolation and expansion protocols, an average of 1.90 × 10^6^ hADSCs from 1 fat pad was obtained. Therefore, the maximum volume repairable using this approach at 5 million cells/ml is estimated to be 380 μl (mm^3^) or 760 μl (mm^3^), using one or two fat pads respectively (Table 3).

**Table 3:** Cartilage defect sizes repairable using minimal hADSCs criteria

	Expanded hADSCs for 5 days → From 1 IFP	Expanded hADSCs for 5 days → From 2 IFPs
Maximum defect repairable (Volume)	380 μl (mm^3^)	760 μl (mm^3^)
Examples of correlating dimensions (Average human knee cartilage depth is 2–4 mm)	3.80 cm^2^ TSA × 1 mm depth	7.60 cm^2^ TSA × 1 mm depth
	1.90 cm^2^ TSA × 2 mm depth	3.80 cm^2^ TSA × 2 mm depth
	1.27 cm^2^ TSA × 3 mm depth	2.53 cm^2^ TSA × 3 mm depth
	0.95 cm^2^ TSA × 4 mm depth	1.90 cm^2^ TSA × 4 mm depth

The calculation was performed using the total number of hADSCs obtained after 5 days of non-passaged expansion and a concentration of 5 million hADSCs/ml. Calculations using cell numbers harvested from 1 or 2 IFPs are shown; furthermore, examples of the extrapolation of correlating dimensions are shown. *TSA* total surface area

### Delivery: stem cell-laden hydrogel therapy in a rabbit *in vivo* cartilage repair model

We then investigated the application of the minimal stem cells concentration identified in our *in vitro* studies, in a rabbit model of cartilage repair using allogenic rabbit adipose derived stem cells. A 4 mm diameter circular defect with a depth of 0.3 mm was chosen, creating a volume of approximately 4 μl (mm^3^). The experimental design was based on previous published literature on rabbit models to test cartilage repair using tissue engineering procedures in critical size defects [47, 48, 49].

Three treatment groups were selected in the study: defect left Empty (Negative control), Microfracture (Positive control) and Therapy (5 million cells/ml laden in hydrogel).

To deliver stem cells-laden hydrogel *in situ* into the cartilage defect, several components of the delivery method require optimisation and design, to enable a user-friendly, efficient, and sterile procedure that is compatible with the surgical environment.

**Fig. 6:**
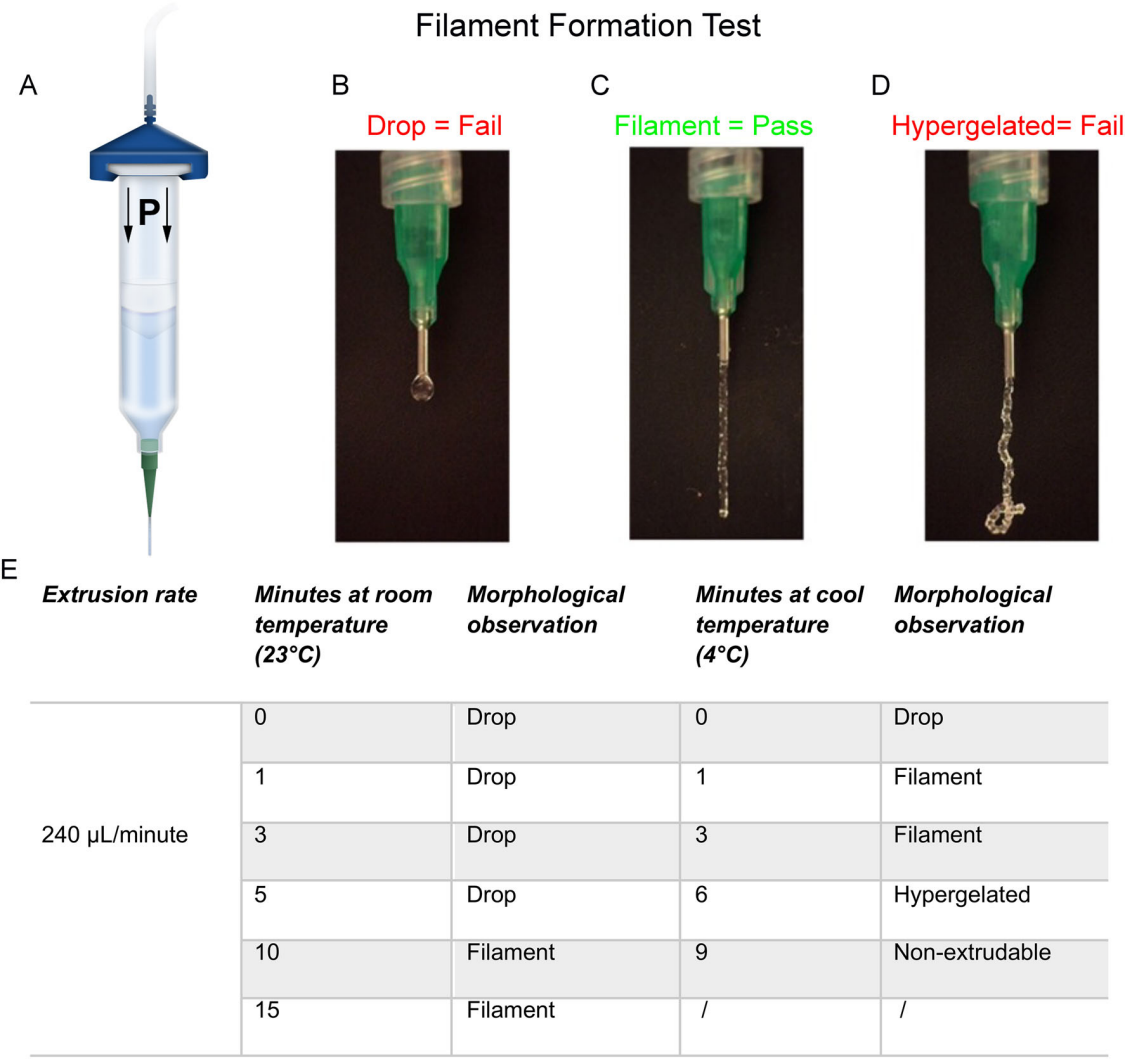
Gelation and extrusion characterization of the hydrogel. **A**–**D** Filament formation test. **A** Graphical representation of the experimental set up. The hydrogel was loaded into 1 ml pneumatic cartridge connected with an 840 µm inner diameter nozzle. The system was kept in an oven at 37 °C to maintain the liquid state for 10 min, and was then allowed to gelate for different time points at 23 °C and 4 °C. After respective gelation times, cartridges were loaded vertically onto the stand of a pneumatic piston device for a filament string formation assay. The pneumatic piston device was set at a constant extrusion rate of 240 μl/min, and the morphology of the hydrogel emerging from the nozzle was observed using a fixed height video recording device. **B**–**D** Representative pictures of the filament extrusion condition when the hydrogel is too liquid and generates drops = fail (**B**), it can form a homogenous filament = Pass (**C**) or it is too viscous so hypergelated = fail (**D**). **E** The table summarizes the properties of the hydrogel being maintained at room temperature (23 °C) or a cool temperature (4 °C) over different time points as measured via filament formation test

First, an efficient gelation time and temperature of the hydrogel used was necessary to ensure a smooth and homogenous delivery and distribution of the stem cells-laden hydrogel inside the cartilage defect. To evaluate the optimal gelation, we used a filament formation test (Fig. 6A–D) [50], where the proxy measure of hydrogel extrusion from the delivery device such as a syringe, is the formation of a filament (string) when extruded from the nozzle, rather than a droplet. By maintaining the hydrogel at 23 °C (room temperature), 10–15 min were required to obtain a string shape extrusion, while by maintaining the hydrogel at 4 °C, only 1–3 min were needed to reach optimal delivery conditions (Fig. 6E). Taking into consideration the heat emanating from the wound in the rabbit, it was decided that 3 min of gelation at 4 °C would be used for the downline *in vivo* model.

**Fig. 7:**
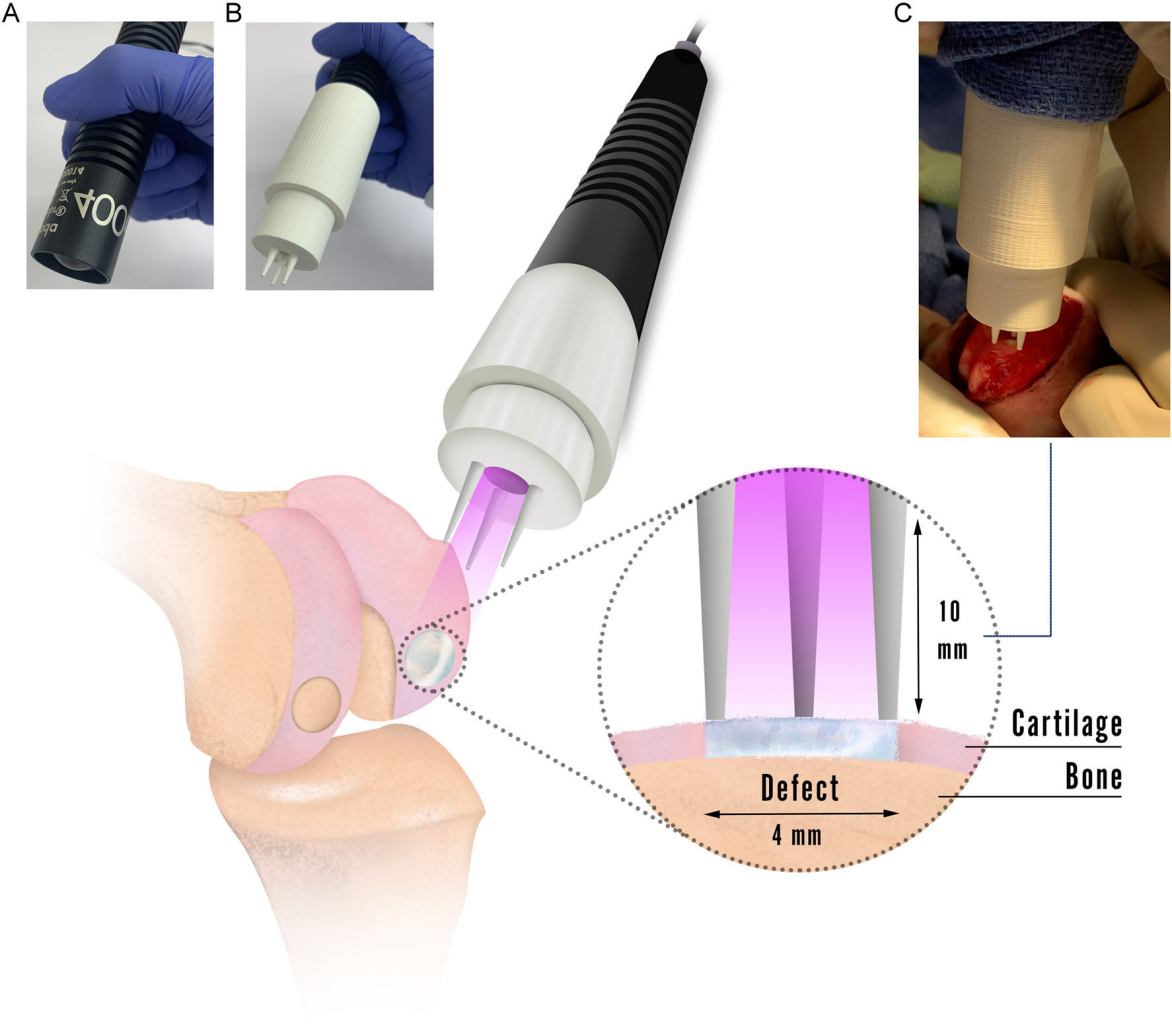
Hydrogel *in situ* light delivery settings. The light source needs to be positioned at 10 mm distance from the cartilage defect to ensure homogenous distribution of the light just over the defect area, and maintain the established intensity of irradiation (20 mW/cm^2^). Therefore, the light probe was equipped with an adapter set-up at a fixed distance of 10 mm for the optimisation of light-defect distance. **A** Photo of the handheld 405 nm light source (LEDsaber, Biolambda, Sao Paulo, Brazil) used for the *in vivo* study. **B** Photo of the fixed distance probe cover adaptor, designed in SOLIDWORKS (Dassault Syst.mes SolidWorks Corporation, Massachusetts, USA) software. The model was then exported in STL format, loaded into Ultimaker Cura (Ultimaker, Utrecht, Netherlands), sliced into G-code using custom settings, and 3D printed using the Ultimaker 3 (Ultimaker, Utrecht, Netherlands) using Polylactic Acid (PLA) (Ultimaker, Utrecht, Netherlands). Before usage, the adaptor was sterilised in ethylene oxide (EtO) gas at room temperature, and then mounted into the LED light enabling comfortable manoeuvring during surgery. **C** Photo of the intraoperative use of the light source and probe cover system, showing how the three prongs gently stabilise the handheld light device

Next, a light-assisted source for cross-linking was set-up to fit within a surgical setting (Fig. 7A). A transition to visible light (405 nm) was undertaken based on emergent findings in the literature and from our team reporting better biocompatibility and penetrance for the hydrogel using visible light compared to UV light [34, 51, 52]. To cover the whole defect without affecting the surrounding native tissue, a 10 mm distance interval was required (Fig. 7) to cover the 4 mm critical defect diameter. Given that a handheld light crosslinking device is most suitable for intraoperative use, a sterilisable attachment probe cover was designed and manufactured via 3D printing (Fig. 7B). The sterilisable 10 mm fixed distance cover was then attached at the end of the light source enabling surgical use (Fig. 7C).

**Fig. 8:**
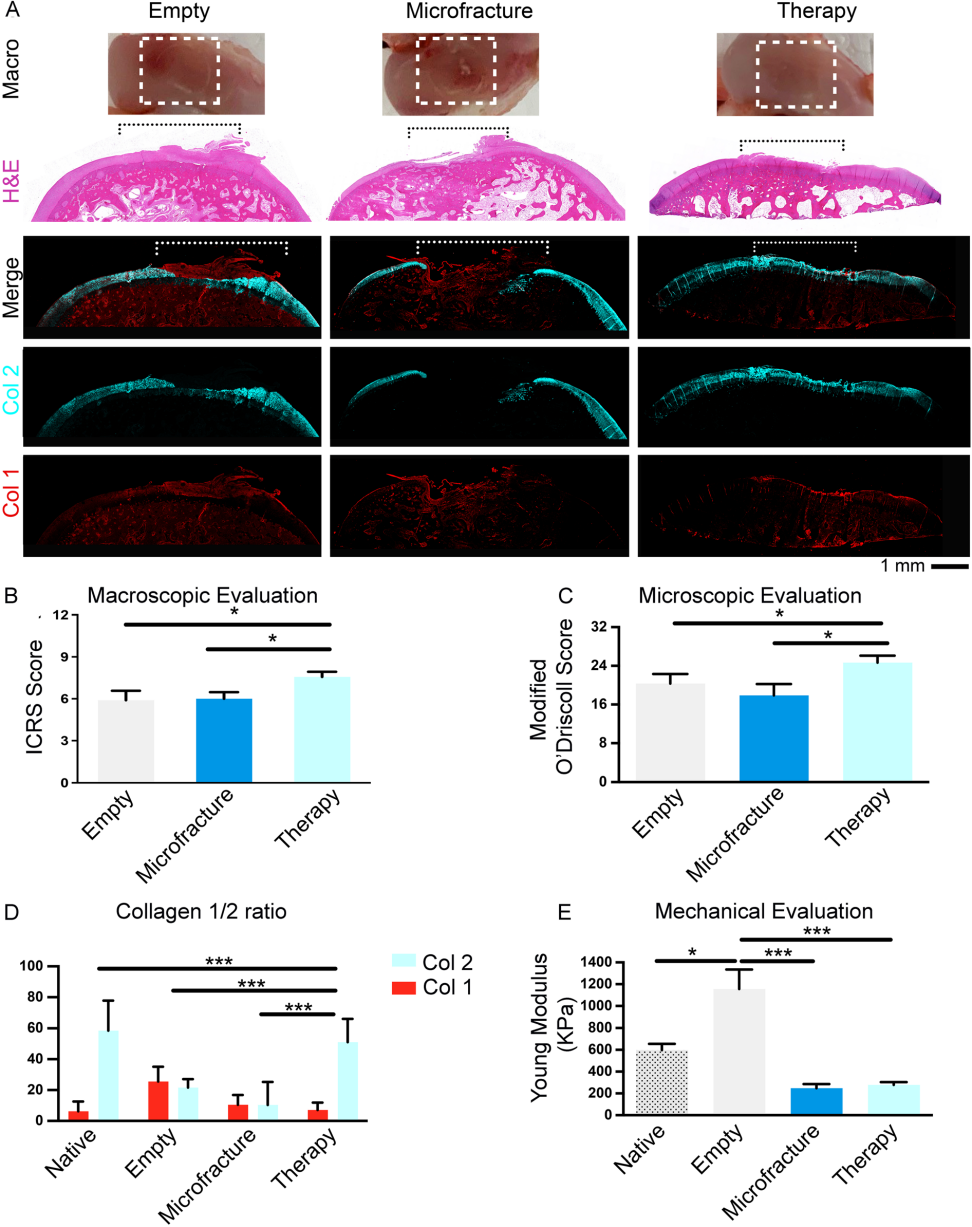
*In situ* stem cells-laden hydrogel therapy in a rabbit *in vivo* cartilage repair model. **A** Representative macroscopic pictures (Macro) and images from Haematoxylin and Eosin (H&E) stained paraffin sections from explanted samples of the indicated groups. Representative confocal images of paraffin sections from explanted samples of the indicated groups assessed using immunostaining for Collagen type 2 (Col 2, in cyan) and Collagen type 1 (Col 1, in red). Overimposed images of the two channels are shown in the Merge raw. **B** The graph shows the macroscopic score using the International Cartilage Repair Society (ICRS) system for the indicated groups, calculated at the end of the 8 weeks study on the explants. **C** The graph shows the microscopic score calculated at the end of the 8 weeks study on the HandE and Col1 and 2 stained paraffin sections. **D** The graph shows the percentage of the Collagen 1 (Col 1) and Collagen 2 (Col 2) positive areas. Graph bars represents the mean with standard deviation of 4 different regions along the entire diameter of the defect for each sample analysed calculated at the end of the 8 weeks study on the immunostained paraffin sections. **E** The graph shows the biomechanical evaluation using atomic force microscopy calculated at the end of the 8 weeks study on the explants and expressed as Young Modulus (kPa kilopascals). Statistical analysis was performed using unpaired t-test

After this optimization phase, the therapy was delivered in the cartilage defect and analyses performed 8 weeks post-surgery. All rabbits survived the study with no preoperative, perioperative, or postoperative complications and were euthanised after 8 weeks. Harvested condyles were visually inspected and evaluated based on the ICRS system to obtain a macroscopic score (Fig. 8). Repair in the therapy (Hydrogel + 5 million cells/ml) group was consistent with a grade II score, associated with nearly normal tissue repair. This was significantly higher than both the empty and microfracture groups (both grades III—abnormal tissue repair) (Fig. 8A and B). From the histological imaging (Fig. 8A), spontaneous regeneration was seen in the empty defect group, with the newly formed tissue looking fibrillated and hypertrophic, with extension beyond the native cartilage surface. The microfracture group showed minimal regeneration, with the fracturing of subchondral bone still visible after 8 weeks. Immunostaining was then performed to evaluate further the type of tissue produced using Collagen type 2 (Col2) as a marker of hyaline cartilage, and Collagen type 1 (Col1) as a marker of scar tissue formation. In the empty group, adequate Col2 expression was evident with a layer of Col1 apparent on top. Col2 in the therapy group was strongly expressed with negligible Col1. A lack of Col2 expression and consistency is observed in the microfracture group with minimal expression of Col1, indicating an absence of tissue regeneration/formation (Fig. 8A). Overall, the therapy group showed regenerative repair and adequate lateral integration to the native tissue.

The microscopic score in the microfracture group was recorded significantly lower than both the empty and therapy groups (Fig. 8C). Interestingly, the ratio between Col2 and Col1 in the therapy treated group was comparable to native cartilage and significantly superior to the ratio scored for the empty and microfracture groups (Fig. 8D).

Finally, the biomechanical properties of the performed treatment groups were assessed using atomic force microscopy (AFM), as described in the methods section. As a reference point, the Young’s modulus of articular knee cartilage in healthy 12 weeks old, male, New Zealand white rabbits is reported to be on average 600 kPa [53, 54]. Consistently, in our AFM analysis the healthy rabbit cartilage (Native) showed a Young’s modulus of 598 ± 57 kPA. After 8 weeks, the empty defects instead showed a Young’s modulus of 1155 ± 179 kPA, significantly higher than both the microfracture and therapy groups and possibly consistent with formation of fibrocartilage, as detected in the Col1 immunostaining performed on the histological sections (Fig. 8A). The therapy group showed a stiffness roughly half that of native cartilage, and although marginally higher than the empty group, no significant difference was scored between them (Fig. 8E).

## Discussion

The field of articular cartilage repair has made significant advances in recent decades [55]. The development of therapies based on stem cells combined with photocrosslinkable hydrogels has open the possibility to translate tissue engineering for articular cartilage repair into clinical practice with the aim of treating cartilage injuries with *in situ* strategies [43, 56, 57]. Nevertheless, several hurdles currently delay the progression of such intraoperative strategies into the clinical settings [5], and satisfactory parameter in terms of cell source, isolation and expansion protocols, delivery parameters, are yet to be standardized [58]. Therefore in our study we highlighted the critical facets required to repair clinically relevant articular cartilage defects using an *in situ* cartilage engineering procedure, the cell-laden hydrogel therapy, where GelMA material is an example of photocrosslinkble hydrogel [43, 59]. The preferable source of cells for articular cartilage regeneration has not yet been identified. Harvesting cells from within the joint that needs to be repaired is desirable compared to utilising a different donor site, since this limits the number and size of surgical incisions and associated risks such as site irritation or infection [60]. Cell types within the knee that have been reported to demonstrate chondrogenic potential include adult chondrocytes, infrapatellar fat pad-derived adipose stem cells and articular progenitor cells [9, 20, 61], defined in our study respectively as hCHOs, hADSCs, and hAPCs. In terms of neocartilage formation efficiency, in this work we found that only infrapatellar fat pad derived stem cells display chondrogenic gene expression and extracellular matrix formation compared to adult chondrocytes and articular progenitor cells. The failure in chondrogenesis observed with chondrocytes and progenitor cells could be attributed to the fact that we used tissue derived from patients undergoing arthroplasty procedure for osteoarthritis, and we were unable to exclude or determine that this was an important factor. The literature suggests there should be no significant effect on chondrogenic capacity in chondrocytes isolated from control patients undergoing ACT treatment with no pre-existing history of osteoarthritis symptoms and, macroscopically healthy cartilage when compared to chondrocytes isolated from patients undergoing total joint knee replacement with severe symptoms of osteoarthritis [62]. However, under our experimental conditions we demonstrated that only the mesenchymal stem cells display chondrogenic potential compared to other cell sources in the intra-articular knee joint area of arthritic patients. Therefore, a comparative analysis of available cells sources for *in situ* cartilage engineering therapy should be carefully evaluated before attempting the clinical translation pathway.

A main advantage of using IFP-derived hADSCs is the possibility of harvesting them from the same site as the subsequent treatment area, spearing healthy cartilage tissue and thereby minimising incisions and surgical risk [60].

The ability to perform cell harvest and implantation within a short time frame and without extensive cells *in vitro* passages, could be of great benefit for clinical translation. The current protocols to isolate and then expand hADSCs require a minimum turnaround of several days, therefore reducing the IFP-derived hADSCs isolation time frame would have major benefits: it avoids any unnecessary exposure of the tissue to enzymatic treatment, while increasing the recovery rate of the stem cell population; it enables a quicker turnaround period of cell culture before reimplantation; it limits the timeframe in contact with animal-derived media and risk of contamination; it decreases the risks associated with long and hazardous procedures associated with *in vitro* expansion. Past attempts at developing rapid hADSCs protocols have failed to adequately isolate and examine a functioning stem cell population with proven chondrogenic potential [63]. In our study we have first developed and validated a rapid 85-min workflow to process hADSCs from IFP tissue, reducing the isolation time respect to our previous protocol where adipose derived stem cells were isolated from IFP in a 3 h chemical digestion time frame [11]. In the current study we used a partially purified preparation of Collagenase type II that according to the manufacturer (Worthington Biochemical Corporation, Lakewood, NJ, USA) is made to contain higher clostripain activity respect to Collagenase type I used in previous studies. We reduced the chemical digestion time from 3 h to 30 min and the overall process was reduced from several days to 85 min, thus reducing the manipulation of the cells, the time, and the overall costs of the manufacturing procedure prior to implantation. Other studies used a timeframe of chemical digestion that spans from 1 to 20 h to obtain adipose derived stem cells from IFP with chondrogenic capacity culture [64, 65, 66]. A limitation of this rapid protocol is the use of Matrigel-coated surfaces to allow selective cellular adherence: although commercially approved for *in vitro* use, this mixture is not approved for clinical use in humans. A synthetically or biologically composition that display the same adhesive capacity needs to be identified for clinical translation [67].

A further prospective improvement to the surgical protocol used in this study, is represented by a minimally invasive, arthroscopic procedure to harvest the IFP. This procedure presents major advantages to save time, reduce cost and minimise morbidity. A successful example is shown in the study by Dragoo and Chang were they successfully isolate adipose derived stem cells from the arthroscopically harvested IFP to provide orthopaedic surgeons with an autologous solution for regenerative procedures [8].

We then extensively validated the cells expansion step post isolation workflow, with the aim to minimize the time required to amplify the number of cells and to eliminate any enzyme-based subculturing step. Recent literature indicates that mesenchymal stem cells can be specifically primed for subsequent chondrogenic differentiation and ECM formation by stimulating cells with FGF2 during the expansion phase [44, 45]. Our data confirmed that expanded IFP derived hADSCs can be used without passaging when primed in a culture media containing EGF and FGF stimulatory growth factors. In our study, we were therefore able to show the earliest turnaround expansion time after the isolation to obtain a minimal number of hADSCs capable to undergo chondrogenesis when stimulated *in vitro*. Our data show that the minimal expansion time without enzyme-based subculturing passaging is 5 days. A key advantage of knowing when the earliest turnaround timeframe for reimplantation can occur is to identify and optimise the patient waiting time between surgical procedures (Harvest and Reimplantation).

Another criterion to define the capacity of the cells to produce neocartilage once embedded into photocrosslinkable hydrogel, is the number of cells per volume of material. As extensively reported by Foldager and co-authors, little is known about how the number of adult chondrogenic cells affects the clinical outcome, and no specific guidelines have been provided in the literature or by regulatory organizations [68]. Therefore, we decided to screen different concentration of cells and score the capacity to produce neocartilage after the production of hydrogel bioscaffolds using photocrosslinked GelMA hydrogel laden with hADSCs. Immunostaining and gene expression analyses, coupled with extracellular matrix production measurement, demonstrated that the 5.0 million hADSCs/ml concentration displays the highest chondrogenic profile and therefore, represents the minimum cell concentration required to trigger the chondrogenic pathway and produce hyaline-like extracellular matrix.

Lastly, we were able to calculate the maximal repairable cartilage defect using the minimal criteria unveiled in terms of cells expansion time and concentration of cells in hydrogel bioscaffolds. The estimated size is 380 μl when 1 IFP is processed, or 760 μl when IFPs from both knees are used. Based on these numbers, all lesions sizes where the microfracture treatment is considered an indication (average volume of 550 μl [69]), can be treated using the proposed tissue engineering procedure. This calculation presented here opens the possibility of personalised repair, which can be individually tailored to each patient based on their defect size.

Several aspects of the delivery procedure of the stem cell-laden hydrogel delivery were also optimised. Using the filament formation test and taking into consideration the surgical room temperature and the patient’s body temperature, it was determined that a gelation time of 3 min at a temperature of 4 °C was best suited to reach an extrudable hydrogel state using a 10% GelMA hydrogel and a syringe as a delivery device. Next, a 10 mm fixed distance handheld visible light crosslinking system set at 405 nm wavelength, was designed utilising a sterilisable attachment cover. This distance ensured that only the defect area of interest was crosslinked, and no healthy tissue was affected in the process.

The final element of this work was to assess the efficiency of the cell-laden hydrogel therapy. A preclinical chondral defect rabbit model was used and a comparison among non-treated defect (Empty), microfracture (clinical standard) and the therapy was performed.

In this regard, it is well recognized that large animal models better approximate human cartilage thickness for *in vivo* testing*.* However, large animal models are expensive and challenging to house [70] therefore, it is well accepted to utilise small animal models like rabbits for proof of concept studies for cartilage repair [71]. Since the early years of cartilage tissue engineering research, the rabbit has been a popular model for osteochondral repair studies because the condyles of mature New Zealand White rabbits are large enough for creation of 3–4 mm defects. This was believed to be a size permitting both the study of new implants and a size where intrinsic repair processes predictably fail. Subsequently, several rabbit studies using chondrogenic cells have demonstrated remarkable endogenous healing potential [72, 73, 74], however, these studies rely on traditional tissue engineering methods using premade constructs combined with flaps or glue [75, 76, 77]. In summary, the rabbit appears to be a practical model for early stages of tissue engineering therapy evaluation, and to test delivery parameters like hydrogel extrusion temperature and intraoperative cross-linking settings, due to relative cost effectiveness, ease of handling, and reasonable joint size for surgical procedures.

After 8 weeks the macroscopic and microscopic assessment showed significantly better results in the stem cell-laden hydrogel therapy treatment group than the empty and microfracture repair with respect to hyaline cartilage formation and quality of the new tissue. Some degree of spontaneous regeneration was seen in the empty treatment group; however, this regeneration was consistent with hypertrophic fibrocartilage production, which is reflected by the significant amount of collagen type 1 in the defect area. In the therapy group, hyaline cartilage regeneration was evident after 8 weeks, with abundant collagen type 2 produced. The biomechanical results obtained with AFM measurements in native rabbit cartilage showed a similar Young's modulus to that reported with 3-month-old male rabbits in the literature measured with unconfined compression test [53] or indentation test [54]. Therefore, AFM was used in our *in vivo* analysis as a measure of the mechanical properties of the articular cartilage, as shown by other authors using rabbit as a preclinical model for cartilage repair [78, 79]. The modulus in the empty group was significantly higher than the therapy and microfracture, and nearly double that of the native cartilage reference, confirming the formation of a stiff fibrocartilage top layer in the empty defect as demonstrated in the microscopic evaluation. Unlike hyaline cartilage, fibrocartilage consists of predominantly type I collagen with minimal GAG content and tendency to ossification that results in greater stiffness compared to articular cartilage [80].

In our study we elucidated the critical facets required to repair clinically relevant articular cartilage defects using an *in situ* tissue engineering procedure defined as cell-laden hydrogel therapy. An efficient 5-Day workflow to rapidly isolate and expand a chondrogenic population of infrapatellar derived adipose stem cells was established. This time frame appears to be sufficient to treat 380 ul volume defect using implantable photocrosslinkable hydrogel at 5milions cells/ml, a concentration capable of producing neocartilage *in vitro*. These minimal criteria together with the optimized delivery paraments, were then validated *in vivo* showing superior hyaline cartilage repair compared to a microfracture technique. The next step is to demonstrate long-term efficacy using a large preclinical animal model, which if successful, will pave the way to human clinical translation. This work presents promise in the future management of chondral defects in young patients with a low-risk strategy that could 1 day treat or halt the progression to early-onset osteoarthritis.

## Supplementary Information

Below is the link to the electronic supplementary material.

Supplementary file1 (DOCX 6753 KB)
