# Supplementary material for: Towards Clinical Translation of In Situ Cartilage Engineering Strategies: Optimizing the Critical Facets of a Cell-Laden Hydrogel Therapy
Source: Tissue Eng Regen Med. 2022 Oct 16;20(1):25–47. doi: 10.1007/s13770-022-00487-9 (PMC9852400; doi:10.1007/s13770-022-00487-9)
Supplement: Supplementary file 1 — Supplementary file1 (DOCX 6753 KB) [file 13770_2022_487_MOESM1_ESM.docx]

**Supplementary Materials and Methods**

**Osteogenic and adipogenic differentiation**

Osteogenic differentiation was induced at P03 by seeding hADSCs in α-MEM supplemented with 2% FBS in six-well plates at 5 × 10^5^ cells per well. The next day, an osteogenic inducing cocktail composed of 10 mM β-glycerophosphate (Sigma, St. Louis, MO, USA), 50 μg/mL ascorbic acid (Sigma) and 100 nM dexamethasone (Sigma) was added. As a negative control, cells seeded under the same conditions were maintained in a non-inducing medium. The medium was changed every 2 days and three biological replicates [n = 3] per time point and type of analysis at 0 and 4 weeks of culture/group were processed. Immuno/histochemistry and Reverse transcription-Quantitative Polymerase Chain Reaction (RT-qPCR) were performed as described below. Relative amounts of RNA were evaluated with the TaqMan Gene expression assay (Applied Biosystems, Foster City, CA, USA) using the following probes: osteocalcin (BGLAP), runX2 and ALP as target genes; GAPDH as the housekeeping gene, details shown in the table below. RT-qPCR was performed using the Quant Studio 6 Flex Real-Time PCR System (Thermo Fisher Scientific), relative quantification was calculated with the 2e^−ΔΔCT^ method. The mean ΔCT value of the control sample was used in each experiment to calculate the ΔΔCT value of sample replicates by using the housekeeping gene (GAPDH).

| **TAQMAN gene markers** | **Applied biosystem reference number** | **Lineage of marker** |
| --- | --- | --- |
| Osteocalcin (BGLAP) | Hs01587814_g1 | Osteogenic |
| RUNX2 | Hs01047973_m1 | Osteogenic |
| Alkaline phosphatase (ALP) | Hs01029144_m1 | Osteogenic |
| Glyceraldehyde 3-phosphate dehydrogenase (GAPDH) | Hs02786624_g1 | House Keeping |

Adipogenic differentiation was induced using cells at passage 3, by seeding hADSCs in six-well plates at a seeding density of 5.0 × 10^5^ cells per well with complete hADSCs culture media. The next day, adipogenic differentiation was commenced using DMEM high-glucose (Sigma), 2% FBS (Gibco), indomethacin 2^-1^ mM, 1 μM dexamethasone, 5^-1^ mM IBMX and 10 μM Insulin Bovine^.^ Adipogenic media was changed twice a week and were collected and frozen at time points of 0 and 3 weeks. Relative amounts of RNA were evaluated with the SYBR power up green Gene expression assay (Applied Biosystems, Foster City, CA, USA) using the following forward and reverse primers: CEBPα and FAB 4 as target genes; GAPDH as the housekeeping gene, details shown in the table below. RT-qPCR was performed using the Quant Studio 6 Flex Real-Time PCR System (Thermo Fisher Scientific), relative quantification was calculated with the 2e^−ΔΔCT^ method. The mean ΔCT value of the control sample was used in each experiment to calculate the ΔΔCT value of sample replicates by using the housekeeping gene (GAPDH).

| **SYBR green gene markers** | **Primer Sequence** | **Lineage of marker** |
| --- | --- | --- |
| CEBPα (CCAAT/enhancer-binding protein alpha) | FORWARD: TATAGGCTGGGCTTCCCCTT  REVERSE: AGCTTTCTGGTGTGACTCGG | Adipogenic |
| FABP4 (Fatty Acid Binding Protein 4) | FORWARD: AACCTTAGATGGGGGTGTCCTG  REVERSE: TCGTGGAAGTGACGCCTTTC | Adipogenic |
| Glyceraldehyde 3-phosphate dehydrogenase (GAPDH) | FORWARD: AATTCCATGGCACCGTCAAG  REVERSE: AGGGATCTCGCTCCTGGAAG | House Keeping |

**Supplementary Figures and Tables**


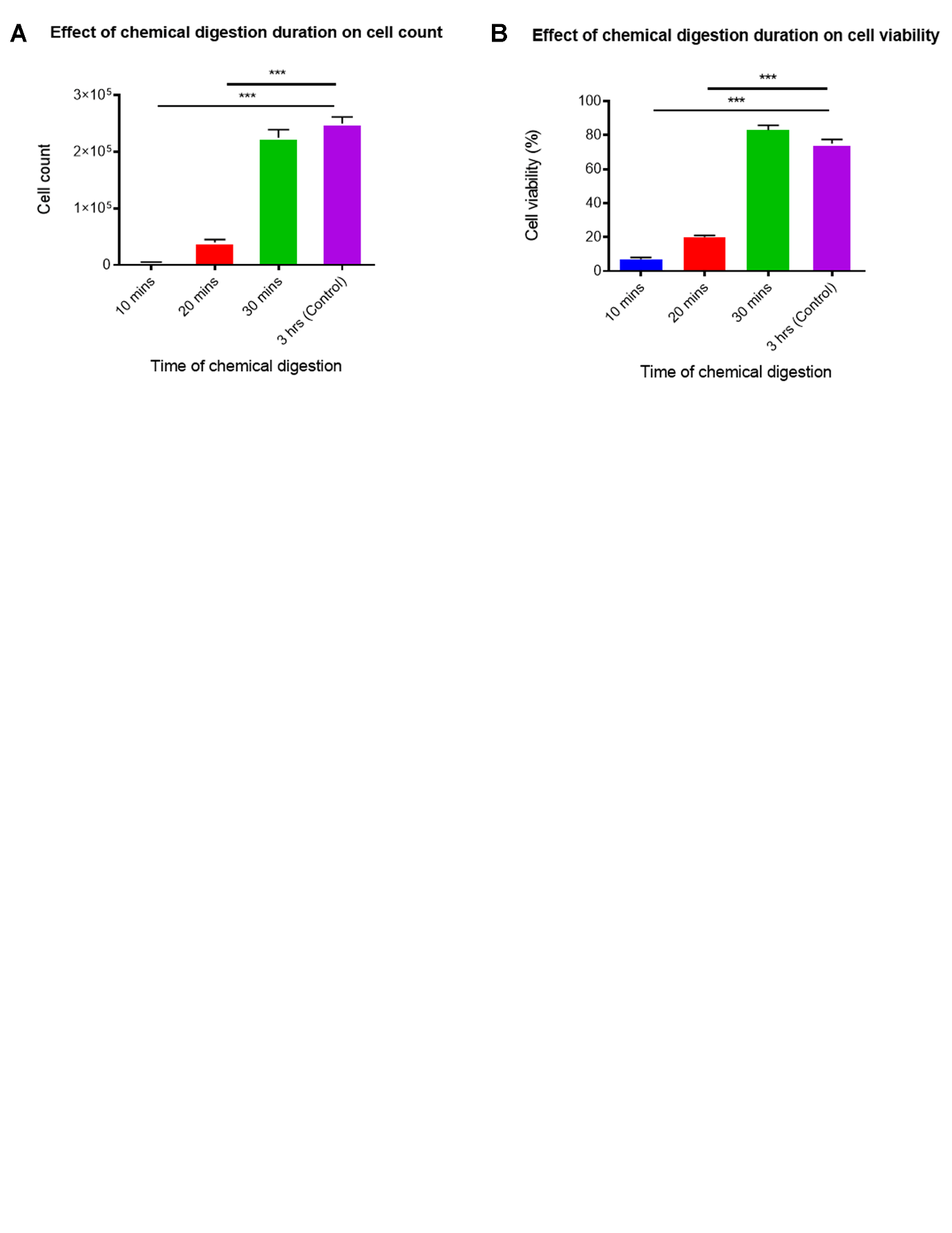


**Figure S1**. **Effect of different durations of enzymatic digestion on cell yield and viability**. Bar graphs representing the effect on cell count (A) and cell viability % (B) after digestion with 345U/ml collagenase type II at the corresponding time points included the standard control 3 hours digestion time frame. The analyses were carried out using trypan blue staining to measure the total cell count and the number of dead cells. Data are presented as mean +/- SEM between three biological replicates from three different patients (n=3). Statistical analysis was performed using unpaired t-test.


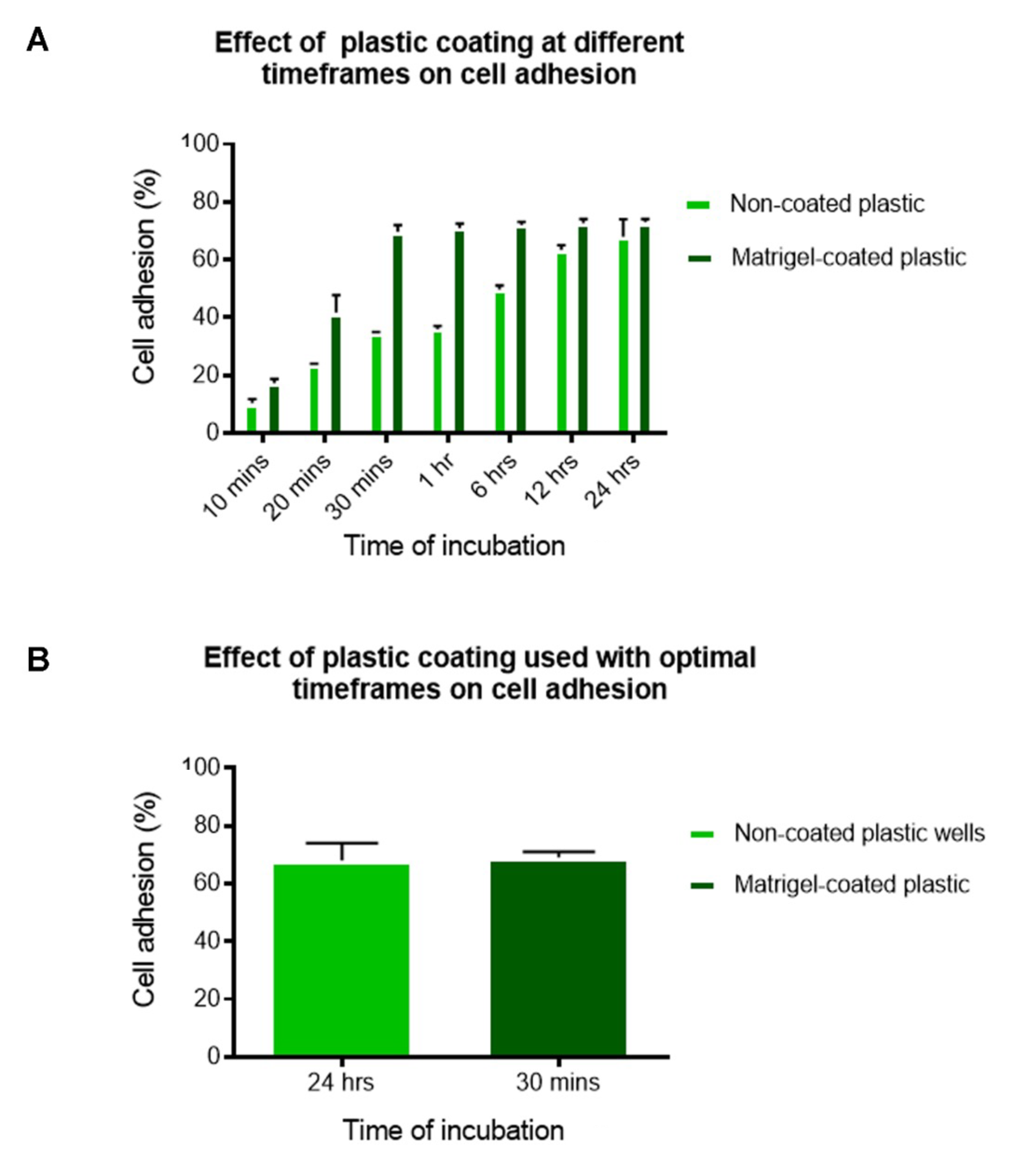


**Figure S2**. **Effect of the duration of non-coated versus Matrigel-coated tissue culture plates on cellular adherence**. (A) Bar graph representing the effect of all timeframes tested. (B) Bar graph representing the direct comparison of non-coated wells at 24 hours compared to Matrigel-coated wells at 30 minutes. The analyses were carried out using trypan blue staining to measure the total cell count and the number of dead cells. Data are presented as mean +/- SEM between three biological replicates from three different patients (n=3). Statistical analysis was performed using unpaired t-test.

 **
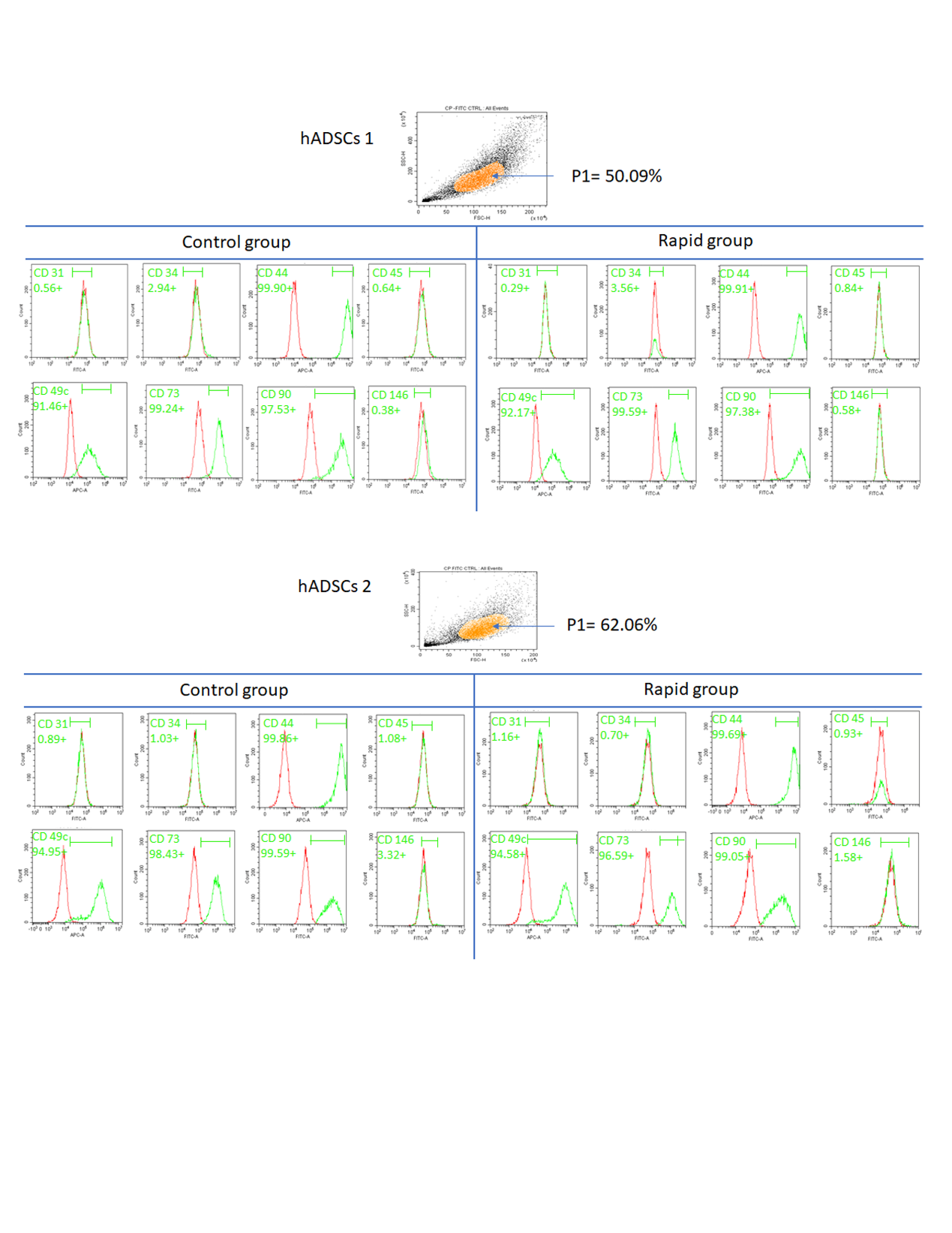
**

**
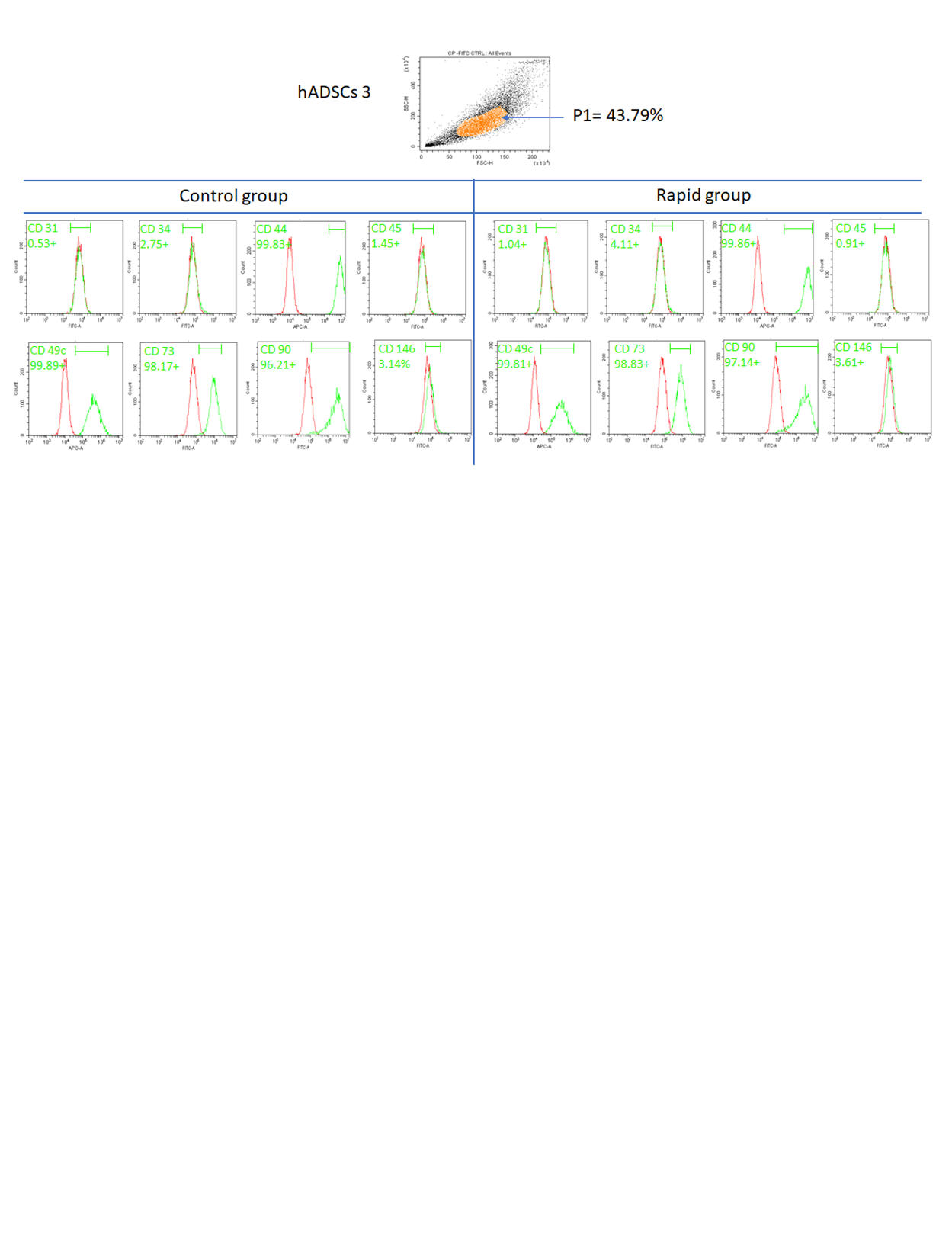
**

**Figure S3. Immunophenotypic characterization using flow cytometry.** The graphs represent the cytofluorimetric analyses of the stemness markers used to characterize the cells in both control and rapid isolation groups in three different hADSCs cell lines for the selected population (P1 in the top graphs). The red profiles represent the control staining with only the secondary antibody, while the green profiles show the expression of the fluorophore-conjugated target antibody (n = 3 independent experiments).


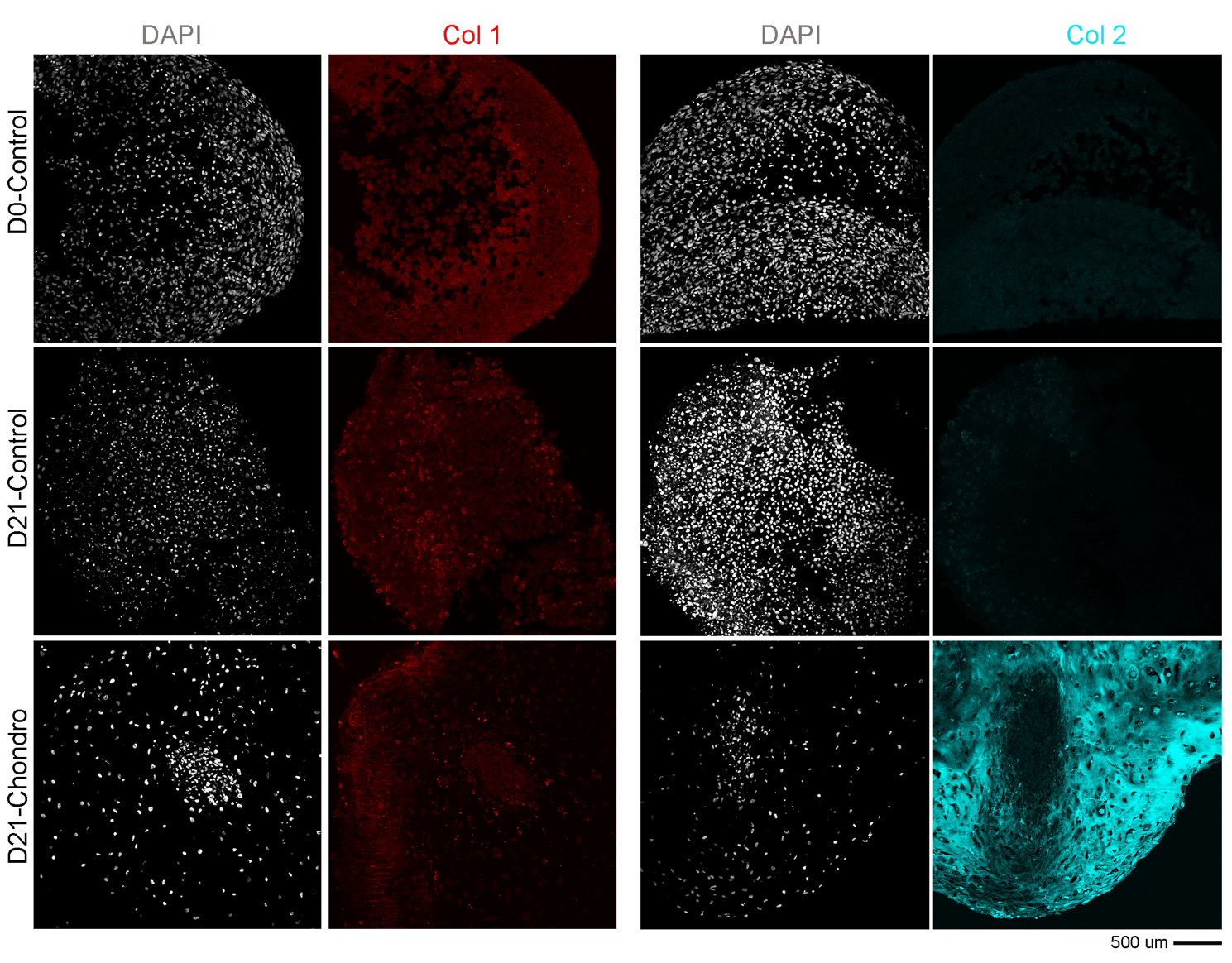


**Figure S4. Chondrogenic analysis in hADSCs pellet culture.** Representative confocal images of cryosections from pellet immunostained with Collagen type 1 (Col 1, in red), Collagen type 2 (Col 2, in cyan) and counterstained to detect cells nuclei (DAPI, in white). For all the stainings, the cryosections were obtained from cells pelleted at day 0 (D0-Control), at day 21 of control samples stimulated with proliferation media (D21-Control), and at day 21 of the chondrogenic stimulation (D21-Chondro).


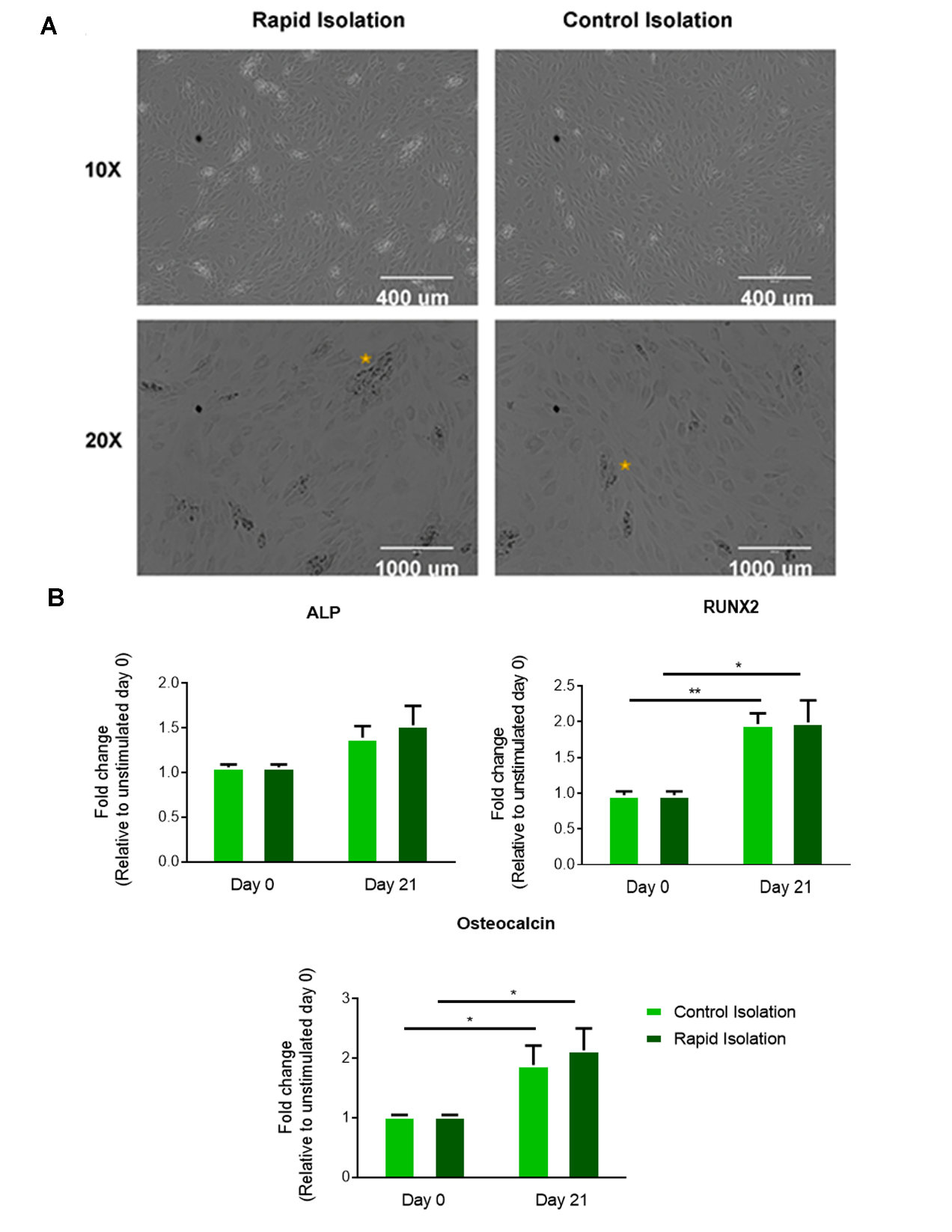
**Figure S5. Osteogenic assessment of rapid and control isolated hADSCs.** (A) Representative brightfield images of cells in 2D monolayer from both control and rapid isolation groups after three weeks of osteogenic differentiation. Objective lens: 10X phase contrast and 20X non-phase. Cell clusters with early mineralisation are highlighted with yellow stars. (B) Osteogenic gene expression analysis: bar graphs represent fold changes relative to day 0 for osteogenic gene markers. Data are presented as mean +/- SEM between three biological replicates from three different patients (n=3). Statistical analysis was performed using unpaired t-test.


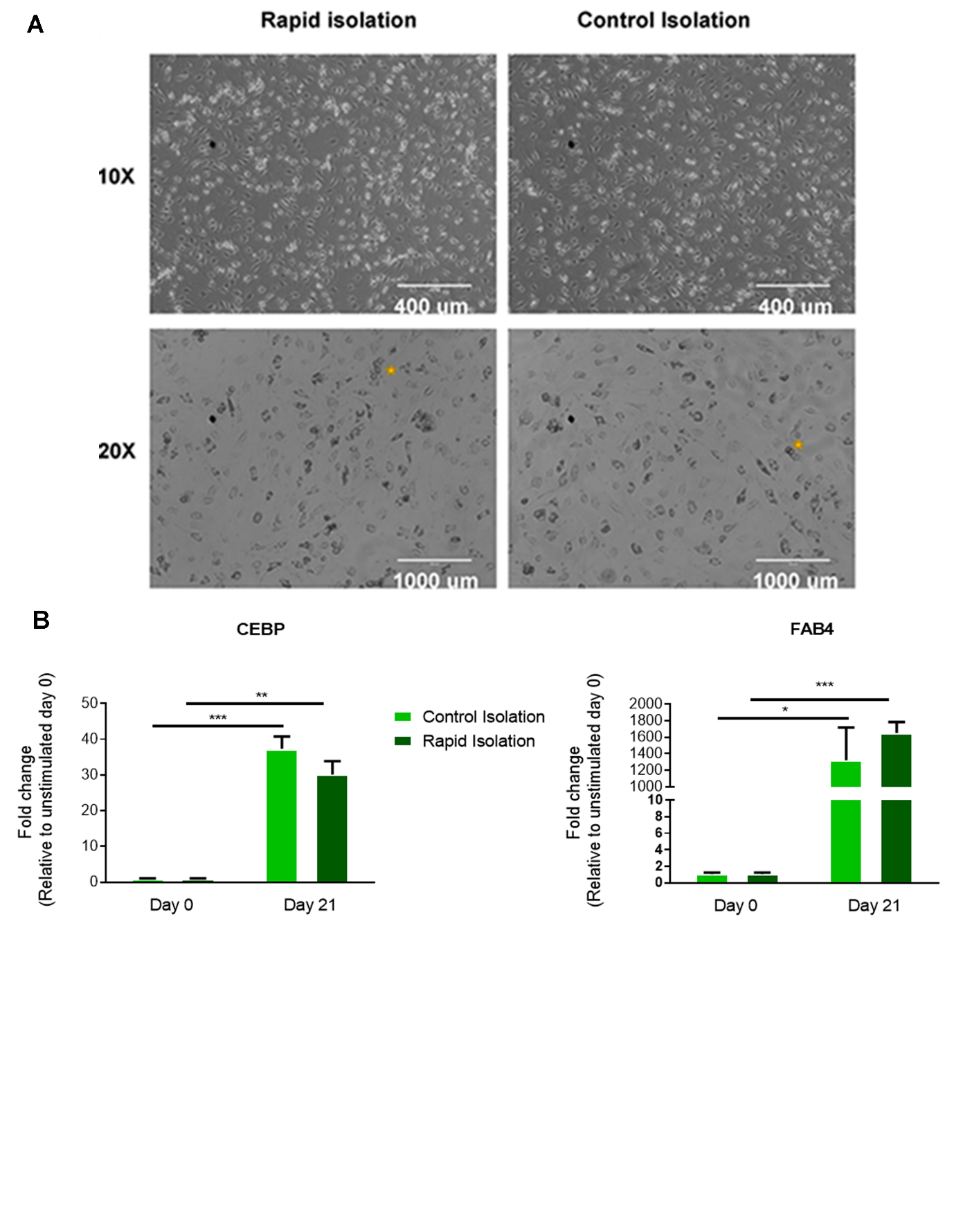


**Figure S6. Adipogenic** **assessment of rapid and control isolated hADSCs.** (A) Representative brightfiled images of of cells in monolayer from both control and rapid groups after three weeks of adipogenic differentiation. Objective lens: 10X phase contrast and 20X non-phase. Micro fat particles/lobules are highlighted with yellow stars. (B) Adipogenic gene expression analysis: bar graphs represent fold changes relative to day 0 for adipogenic gene markers. Data are presented as mean +/- SEM between three biological replicates from three different patients (n=3). Statistical analysis was performed using unpaired t-test.


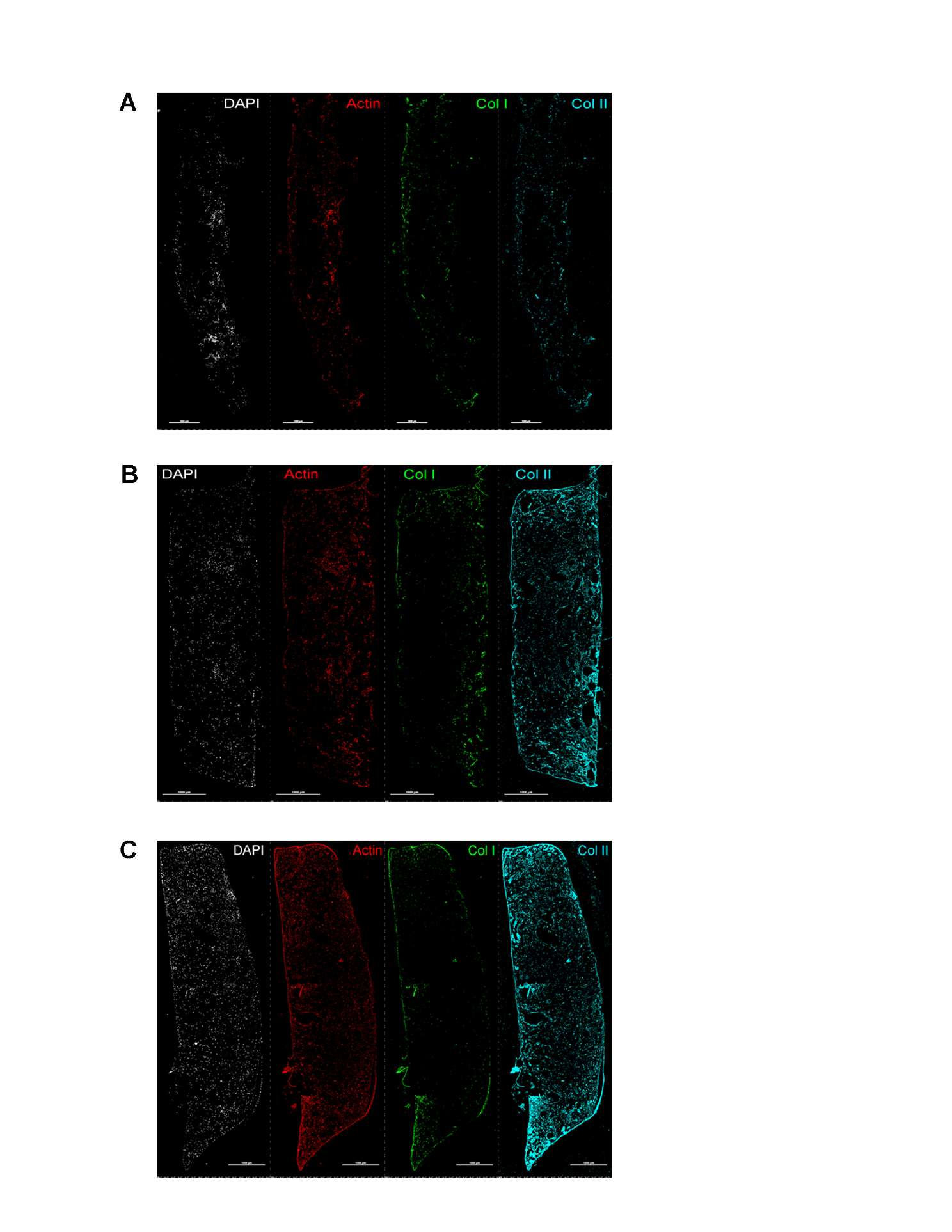


**Figure S7. Assessment of minimal hADSCs concentration required for chondrogenesis in hydrogel bioscaffolds: imaging analysis.** Representative confocal microscopy images of entire cryosections from bioscaffolds at the three different cell concentrations after 3 weeks of chondrogenesis. The cryosections has been obtained by cutting the samples along the z axis to provide spatial information from the top (on the left side of the panels) to the bottom (on the right side of the panels) of the bioscaffold.

(A) 1.25 million hADSCs/ml concentration group (B) 2.5 million hADSCs/ml concentration group C) 5.0 million hADSCs/ml concentration group. Figure legend: DAPI – Cell nucleus, Actin – cytoskeleton, Col I – Collagen type 1 and Col II – Collagen type 2.


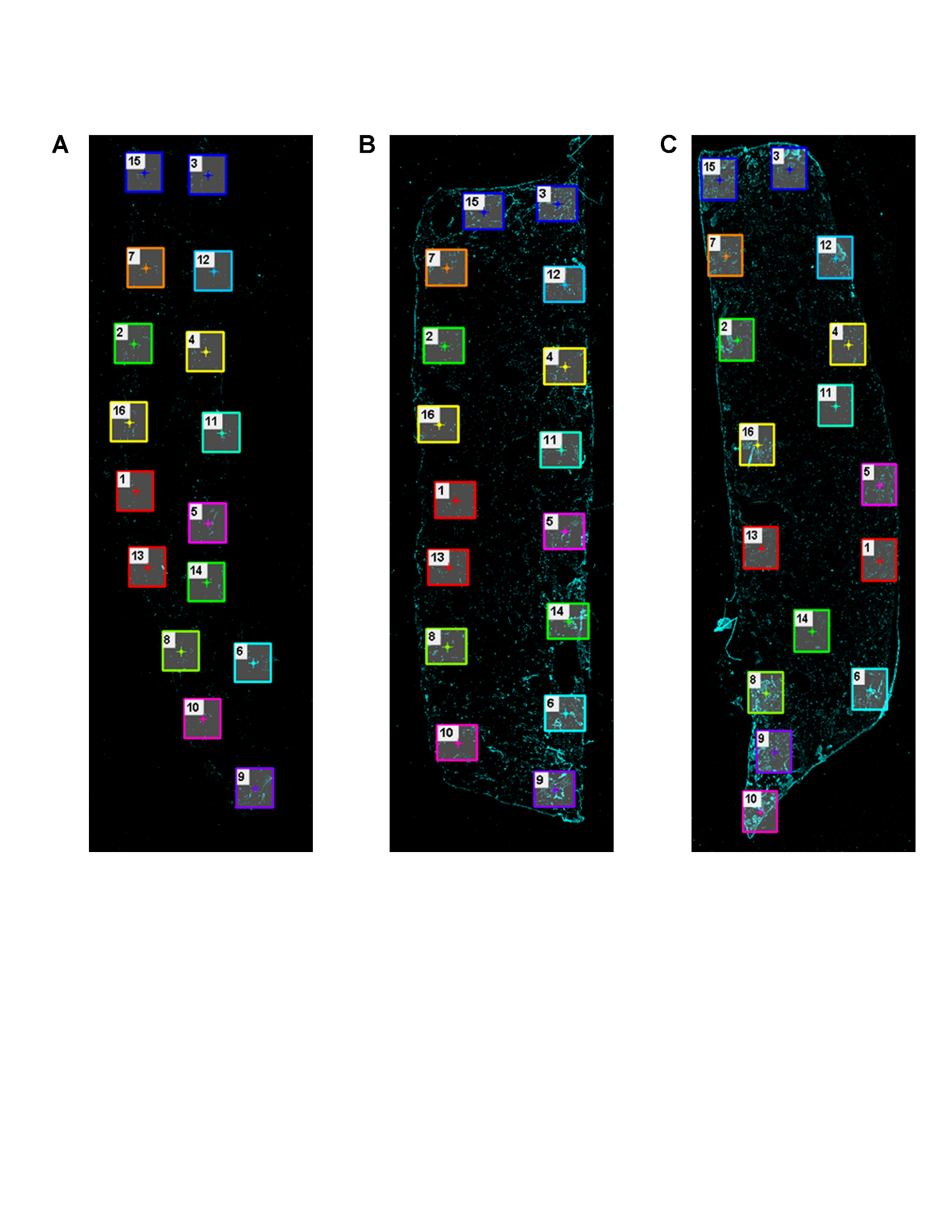

**Figure S8. Assessment of minimal hADSCs concentration required for chondrogenesis in hydrogel bioscaffolds: Collagen type 2 intensity quantification analysis.** Representative images of cryosections from (A) 1.25 million hADSCs/ml concentration group (B) 2.5 million hADSCs/ml concentration group (C) 5.0 million hADSCs/ml concentration group bioscaffolds after 3 weeks chondrogenesis. The regions of interest (ROI) used for the calculation of Collagen 2 (Col 2) staining intensity (related to Figure 5C) are shown.16 ROI square boxes were used in all samples. Staining indicating Collagen 2 accumulation is visible in cyan (n = 2 independent experiments).
